# Sustained Transcriptional Response to Lipopolysaccharide and Interleukin-4 in an Immortalized Mouse Microglial Cell Line

**DOI:** 10.1007/s12035-026-05711-4

**Published:** 2026-02-06

**Authors:** Amanda Herrero-González, Alba Puente-Sanz, Diego Pérez-Rodríguez, Berta Anuncibay-Soto, Michal Letek, Marta Regueiro-Purriños, Arsenio Fernández-López

**Affiliations:** 1 Departamento de Biología Molecular, Universidad de León, León, Spain; 2 Neural Therapies S.L., Edificio Institutos de Investigación, Local B43. Campus de Vegazana, León, Spain; 3 Departamento de Medicina, Cirugía y Anatomía Veterinaria, Universidad de León, León, Spain; 4 Dipartimento Di Scienze Della Salute, Universitá Di Firenze, Florence, Italy; 5 Department of Clinical and Movement Neurosciences, UCL Queen Square Institute of Neurology, London, UK; 6 Department of Life Sciences, Imperial College London (ICL), London, UK

**Keywords:** Microglial responses, Pro-inflammatory stimuli, Anti-inflammatory stimuli, RNA-seq, Sustained expression genes

## Abstract

In an attempt to identify markers that better characterize microglial states and to search for potential therapeutic targets, we performed a study using the IMG cell line as an in vitro microglial model. Specifically, we tested its response to several pro-inflammatory stimuli (lipopolysaccharide, interferon gamma, and tumor necrosis factor) and an anti-inflammatory stimulus (Interleukin-4) after 12 and 24 h of incubation. We performed RNA sequencing to identify genes modified at both incubation times (i.e., genes with sustained changes in the window between 12 and 24 h) that could reflect sustained microglial transcriptional responses. We also used Gene Ontology (GO) analysis to identify the most relevant pathways modified by these stimuli. RNA sequencing revealed four gene sets: **(**1) common genes that respond similarly to IL-4 and LPS, (2) specific LPS responders, (3) specific IL-4 responders, and (4) genes that exhibit LPS-induced upregulation and IL-4-induced downregulation, and vice versa (opposite responders). We hypothesize that the common gene set represents a general microglia response to pathological conditions, while the LPS- and IL-4-responder gene sets define specific microglial states under pro- and anti-inflammatory stimuli, respectively. We further propose that opposite responder genes act as metabolic switches between certain microglial states. The GO analysis indicated that LPS strongly upregulates biological processes related to the innate immune response, while IL-4 upregulates pathways related to repair, metabolic reprogramming, and cellular cooperation. Finally, the transcriptional response of IMG cells closely mirrored that of primary microglia, revealing highly similar gene expression and GO term profiles under LPS stimulation.

**Graphical Abstract:**

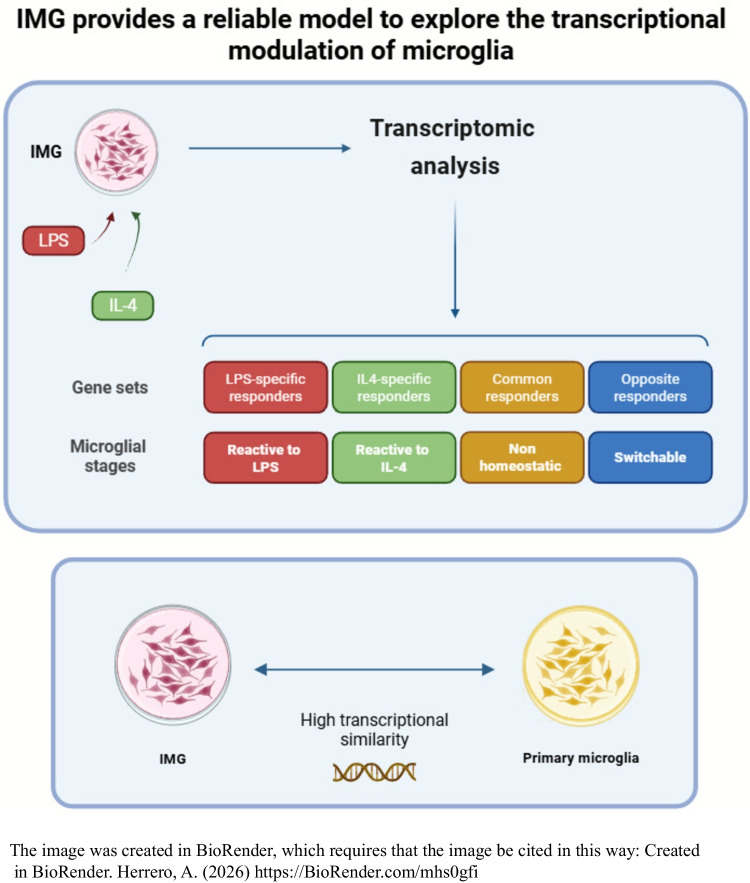

**Supplementary Information:**

The online version contains supplementary material available at 10.1007/s12035-026-05711-4.

## Introduction

Microglia perform a wide range of functions in the central nervous system (CNS), including the regulation of brain development, the maintenance of neuronal networks, episodic memory, and injury repair [1–5]. Microglia maintain brain homeostasis through local self-renewal under normal physiological conditions. They are the central drivers of the inflammatory processes in CNS pathologies, and their modulation is crucial to fight neurodegenerative diseases such as Alzheimer’s disease (AD), Parkinson’s disease, multiple system atrophy, amyotrophic lateral sclerosis, frontotemporal dementia, progressive supranuclear palsy, corticobasal degeneration, dementia with Lewy bodies, and Huntington’s disease [6–8]. The inflammatory evolution is also closely linked to the progress of other pathologies with high prevalence, such as stroke and traumatic brain injury [9, 10].

Researchers have developed and tested several microglial murine cell lines. In particular, BV2 cells have been widely used to characterize the properties of microglia in vitro [11, 12]. While this cell line has been used as a substitute for primary microglia, concerns have been raised about its suitability [13–15]. For example, lipopolysaccharide (LPS) induces ten-fold more genes in primary microglia compared with BV2 cells [16]. IMG cells are a more recently described microglial cell line. This line was derived from the adult mouse brain and has been immortalized by retrovirus v-raf/v-myc. It expresses the microglial markers CD11b and F4/80 and has been reported to present strong responses to both pro- and anti-inflammatory stimuli [17]. IMG cells respond to LPS stimulation by upregulating inducible nitric oxide synthase (iNOS, encoded by *Nos2*), tumor necrosis factor alpha (TNFα, encoded by *Tnf*), and interleukin 1beta (IL-1β, encoded by *Il1b*) from 8 to 24 h, and respond to IL-4 by upregulating *Arg1*, *Mgl1*, and *Ym1* from 8 to 24 h [17]. Incubation of IMG cells with LPS induces rapid activation of the NLRP3 inflammasome, detectable as early as 4 h, although this response is not sustained at 24 h [18]. Activation of IMG cells by amyloid beta, a hallmark of AD, highlights its utility as a valuable in vitro model for studying neurodegenerative disorders [17].

The classical terminology has used the term M0 to describe resting microglia and describes a detrimental M1 phenotype, characterized by the activation of pro-inflammatory pathways, or a beneficial M2 phenotype, described as supporting tissue repair, debris clearance, and inflammation resolution. The M2 phenotype, in turn, has been subdivided into M2a microglia, activated by IL-4 or IL-13; M2b microglia, which arise when Fc receptors engage immune complexes in LPS- or IL-1β-primed cells; and M2c microglia, induced by IL-10 or glucocorticoids [11, 19–24].

This dichotomic view of the “bad and good” microglial states has been challenged in the last years. A more recent perspective considering a wide number of microglial states and functions has emerged, with a recommendation to avoid the terms activated and inactivated microglia and the M1 and M2 labels [25, 26]. These conflicting views hinder researchers from establishing more meaningful microglial polarization pathways and terminology. Therefore, a careful revision of the microglial polarization concept is needed to advance the field [27]. Different functional states have been described in response to pro-inflammatory and anti-inflammatory stimuli. In an attempt to contribute to the characterization of these functional states, we present a transcriptomic study covering the 12- to 24-h time window following exposure to either pro-inflammatory or anti-inflammatory conditions. These states are described to reflect different functional states, which should be mirrored by their transcriptomic profiles. In this study, we found that a subset of genes presented a sustained expression at both 12 and 24 h under either stimulus. Therefore, these upregulated genes activated by both LPS and IL-4 mirror the loss of homeostatic response rather than stimulus-specific functional responses to the inflammatory or anti-inflammatory stimuli. In this regard, we describe for the first time the following gene sets: specific LPS-responder genes, specific IL-4-responder genes, common LPS- and IL-4-responder genes, and opposite genes—genes that are upregulated by LPS and downregulated by IL-4 expression, and genes that are downregulated by LPS and upregulated by IL-4 expression. Moreover, we used Gene Ontology (GO) analysis to show the divergence in the differentiation pathways elicited by LPS and IL-4 in the IMG model.

## Material and Methods

### Reagents

The immortalized mouse microglial cell line (IMG) was obtained from Millipore (#SCC134; Burlington, MA, USA). The cells were cultured in high-glucose (4.5 g/L) Dulbecco’s Modified Eagle Medium (DMEM; #21969035), supplemented with 10% fetal bovine serum (FBS; #A5256701, Lot#: 2575633), 2 mM L-glutamine (#25030032), and 100 U/mL penicillin-streptomycin (P/S; #15140122), all from Gibco (Thermo Fisher Scientific, Waltham, MA, USA), here referred to as “culture medium.” For cell subculture, 0.05% trypsin-EDTA (#25,300,062; Gibco) was used.

Recombinant murine TNFα (#31501A100UG), interferon gamma (IFNγ; #31505100UG), and IL-4 (#214145UG) were purchased from Gibco (Thermo Fisher Scientific). LPS (*Escherichia coli* O111:B4; #L2630) was obtained from Sigma-Aldrich (St. Louis, MO, USA).

RNA was extracted by using the Tripure Isolation Reagent (#11667165001; Roche Diagnostics, Basel, Switzerland) or the E.Z.N.A.® Total RNA Kit I (#R6834-00S; Omega Bio-Tek, Norcross, GA, USA). Reverse transcription (RT) of RNA into first-strand complementary DNA (cDNA) synthesis was carried out using the PrimeScript™ RT Reagent Kit (#RR037A), and quantitative polymerase chain reaction (qPCR) was performed using TB Green® Premix Ex Taq™ (#RR420W), both provided by Takara Bio Inc. (Kusatsu, Shiga, Japan).

IL-1β levels in culture supernatants were quantified by using a mouse IL-1β enzyme-linked immunosorbent assay (ELISA) kit (#KE10003; Proteintech, Rosemont, IL, USA). Nitric oxide (NO) production was assessed with the Griess Reagent Kit (#G7921; Invitrogen, Thermo Fisher Scientific, Waltham, MA, USA).

### Cell Culture and Treatments

The cells were seeded in 12-well plates at a density of 2.5 × 10^5^ cells/well and cultured at 37 °C in a humidified atmosphere containing 5% CO_2_. After 24 h, they were stimulated with increasing concentrations of LPS, IFNγ, or TNFα (10, 50, and 100 ng/mL), or with IL-4 (10, 25, and 50 ng/mL). Untreated cells served as the control condition. After incubation for 12 or 24 h, cells were harvested for RNA extraction and downstream analysis by RT-qPCR and RNA sequencing, while culture supernatants were collected for protein quantification. There were six experimental conditions for transcriptomic analysis: **(**1) incubation in 100 ng/mL LPS for 12 h (12LPS); (2) incubation in 25 ng/mL IL-4 for 12 h (12IL4); (3) incubation only with culture medium for 12 h (12C), which was used as a control for the 12LPS and 12IL4 conditions; (4) incubation in 100 ng/mL LPS for 24 h (24LPS); (5) incubation in 25 ng/mL IL-4 for 24 h (24IL4); and (6) incubation only with culture medium for 24 h (24C), which was used as a control for the 24LPS and 24IL4 conditions. Five independent experiments were performed for RNA sequencing.

### RNA Analysis

After incubation, the cells were collected and stored at −  80 °C until further processing. For qPCR, total RNA was extracted using the TriPure Isolation Reagent, following the manufacturer’s protocol. For transcriptomic analysis, total RNA was isolated from frozen cell pellets by using the E.Z.N.A.® Total RNA Kit I, according to the manufacturer’s instructions.

### qPCR

Dose–response assays and RNA sequencing (RNA-seq) validation were performed using quantitative real-time PCR (RT-qPCR) assays, following the Minimum Information for Publication of Quantitative Real-Time PCR Experiments (MIQE) guidelines [28]. Total RNA was extracted using the Tripure reagent, and its concentration and purity were assessed with a NanoDrop spectrophotometer (Thermo Fisher Scientific). RNA integrity was evaluated by electrophoresis in 1% agarose gels. Samples were excluded if the A260/280 ratio was below 2.0 or if electrophoresis did not reveal the presence of two distinct ribosomal RNA bands. cDNA was synthesized from high-quality RNA by using the PrimeScript RT Reagent Kit. Primer sequences used for qPCR are listed in Table 1 and were designed with the Primer-BLAST tool (National Center for Biotechnology Information (NCBI)).

**Table 1:** Primer information used for quantitative real-time polymerase chain reaction. This includes the NCBI RefSeq accession numbers, the forward and reverse sequences, the amplicon size, and the amplification efficiency

Gene	NCBI RefSeq	Forward (5′ → 3′)	Reverse (5′ → 3′)	Product length (pb)	Efficiency (%)
*Arg1*	NM_007482.3	CATTGGCTTGCGAGACGTAGAC	GCTGAAGGTCTCTTCCATCACC	124	93.98
*H3f3a*	NM_008210.6	CGCTTCCAGAGTGCAGCTATT	ATCTTCAAAAAGGCCAACCAGAT	72	105.46
*Igf1*	NM_010512.5	TTCTACCTGGCGCTCTGCTT	AGCCTGTGGGCTTGTTGAAGT	139	108.97
*Il1b*	NM_008361	TGGACCTTCCAGGATGAGGACA	GTTCATCTCGGAGCCTGTAGTG	148	106.43
*Mrc1*	NM_008625.2	GGAGTGATGGTTCTCCCGTTT	ACATGCCAGGGTCACCTTTC	105	94.56
*Nfkbia*	NM_010907.2	CTGGCCAGTGTAGCAGTCTT	GACACGTGTGGCCATTGTAG	91	92.33
*Nos2*	NM_010927.4	GGTGAAGGGACTGAGCTGTT	CCAACGTTCTCCGTTCTCTTG	106	109.05
*Ptgs2*	NM_011198.5	TGAGTACCGCAAACGCTTCT	CAGCCATTTCCTTCTCTCCTGTA	74	90.59
*Tgfb1*	NM_011577	TGATACGCCTGAGTGGCTGTCT	CACAAGAGCAGTGAGCGCTGAA	107	110.89
*Tnf*	NM_013693.3	GTCCCCAAAGGGATGAGAAGTT	GCTACAGGCTTGTCACTCGAA	109	93.34

*Arg1*, arginase-1; *H3f3a*, H3.3 histone A; *Igf1*, insulin-like growth factor 1; *Il1b*, interleukin-1 beta; *Mrc1*, mannose receptor, c-type 1; *Nfkbia*, nuclear factor of kappa light polypeptide gene enhancer in B-cell inhibitor, alpha; *Nos2*, nitric oxide synthase 2; *Ptgs2*, prostaglandin-endoperoxide synthase 2; *Tgfb1*, transforming growth factor beta 1; *Tnf*, tumor necrosis factor

qPCR was carried out in a StepOnePlus™ Real-Time PCR System (Applied Biosystems, Waltham, MA, USA). Each reaction contained 9 µL of TB Green® Premix Ex Taq™ containing 300 nM of each primer and 1 µL of a 1:10 dilution of the reverse transcription product (corresponding to 600 ng of cDNA). The thermal cycling conditions were as follows: initial denaturation at 90 °C for 30 s, followed by 40 cycles of 5 s at 95 °C and 30 s at 60 °C, and a final melting curve analysis. Primer specificity was confirmed by melting curve analysis and agarose gel electrophoresis. Amplification efficiency was determined from standard curves generated using serial dilutions of cDNA (Table 1). The relative transcript levels were calculated using the 2^−ΔΔCt^ method [29] with *H3f3a* (which encodes H3.3 histone A) used as the reference gene. The qPCR results were confirmed in at least three independent experiments.

### RNA Sequencing

RNA sequencing was performed by Novogene Co., Ltd. (Beijing, China). In brief, total RNA quality was assessed with a Qubit, a NanoDrop, agarose gel electrophoresis, and an Agilent BioAnalyzer. Messenger RNA (mRNA) was enriched using oligo(dT)-coated magnetic beads, fragmented, and reverse transcribed using random hexamer primers. Second-strand cDNA synthesis was performed with either dUTP (directional libraries) or dTTP (non-directional), following Parkhomchuk et al. [30]. Library preparation included end repair, A-tailing, adapter ligation, size selection, and PCR amplification, with USER enzyme digestion added for directional libraries. The libraries were quantified by Qubit (Thermo Fisher) and qPCR, size-checked with an Agilent 2100 Bioanalyzer (Agilent Technologies, Santa Clara, CA, USA), and pooled equimolarly before sequencing on an Illumina NovaSeq platform (PE150) (Illumina, San Diego, CA, USA). Raw FASTQ data were filtered by Novogene to remove low-quality reads, adapter sequences, and reads with > 10% “N.”

### Bioinformatic Analysis

Raw reads (fastq format) were processed through in-house Perl scripts, and paired-end clean reads were aligned to the reference genome using Hisat2 v2.05 [31]. The mapped reads of each sample were assembled by StringTie v1.3.3b [32] in a reference-based approach, and FeatureCounts v1.5.0-p3 [33] was used to count the read numbers mapped to each gene. Principal component analysis (PCA) and differential expression analysis were performed using the DESeq2 R package (1.20.0) [34].

Volcano plots were generated with R filtering for adjusted *p*-value < 0.05 (horizontal dotted line) and log_2_ fold changes >  + 1 and < –1 (vertical dotted line). Venn diagrams were generated with the ggvenn R package (v 0.1.16) [35] (10.32614/CRAN.package.ggvenn). GO enrichment analysis was performed using the enrichGO function from the clusterProfiler R package [36], in combination with resources from the Bioconductor R package [37]. The annotation libraries org.Mm.eg.db (for mouse genes) and org.Hs.eg.db (for human genes) were used to identify orthologous relationships between species [38]. Only GO terms with an adjusted *p*-value < 0.05 were considered to be significantly enriched.

**Fig. 1:**
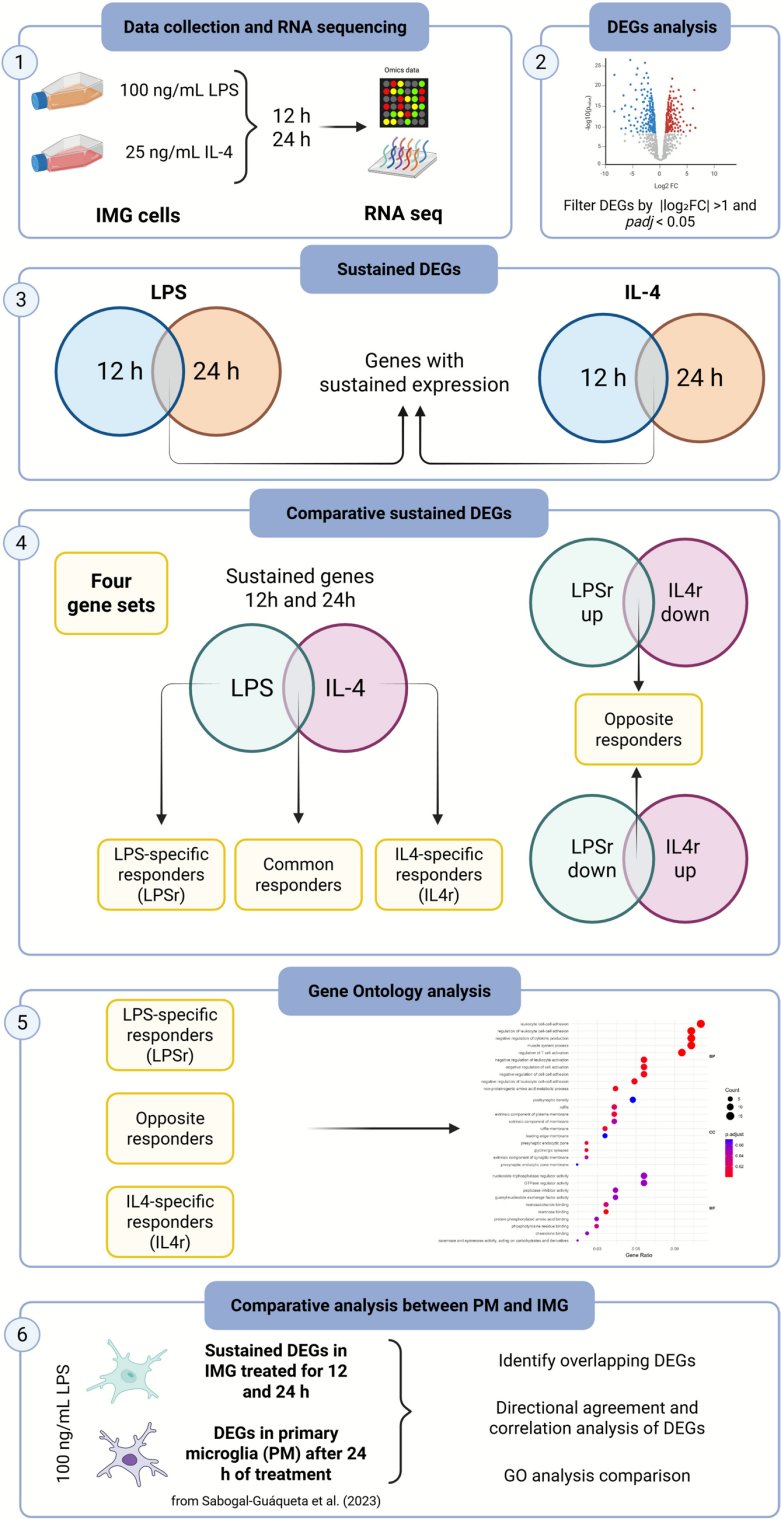
**RNA-seq sequencing workflow and analysis overview**. Experimental workflow for RNA-seq based on differential expression gene (DEG) analysis in stimulated IMG cells. (1) Cells were treated with 100 ng/mL LPS or 25 ng/mL IL-4 for 12 and 24 h, and RNA was collected for sequencing. (2) DEGs were identified and filtered by |log_2_FC|> 1 and *p*_adj_ < 0.05. (3) Sustained DEGs were defined as genes regulated at both 12 h and 24 h. (4) Comparative analysis of sustained DEGs identified four gene sets: LPS-specific responders (LPSr), IL-4–specific responders (IL4r), common responders, and opposite responders. (5) GO analysis was performed on LPS- and IL-4–associated DEG sets (excluding common responders) to reveal functional signatures that distinguish LPS- vs. IL-4-driven programs; opposite responders are included within these LPS- and IL-4–associated sets according to directionality. (6) Comparative analysis between IMG_sustained_ gene set and primary microglia (PM; dataset from Sabogal-Guáqueta et al. [39]) was performed to identify overlapping DEGs, assess directional agreement and correlation, and compare GO enrichment profiles. Created in BioRender. Herrero, A. (2026) https://BioRender.com/lnivk11

Figure 1 illustrates the RNA-seq workflow applied in this study, including the bioinformatic analysis for differential expression analysis, the identification of sustained and condition-specific DEGs, and the subsequent GO enrichment analysis.

### Comparison Between Sustained IMG and Primary Microglia (PM) in Pro-inflammatory Conditions

To validate our IMG model, we compared the transcriptional response of immortalized microglia (IMG) after 24 h of LPS stimulation (100 ng/mL) with that of primary mouse microglia (PM) subjected to the same treatment, obtained from the publicly available dataset of Sabogal-Guáqueta et al. [39] (GEO accession number GSE221013).

We validate only the subset of differentially expressed genes (DEGs) from the IMG model that remained significantly differentially expressed at both 12 h and 24 h following LPS exposure, which we defined as sustained DEGs (IMG_sustained_) for this time frame. DEGs from the IMG_sustained_ and PM datasets were identified by comparing fold changes relative to control conditions using DESeq2 (v1.38) [40] and applying identical significance thresholds (adjusted *p* < 0.05 and |log₂FC|> 1). The IMG_sustained_ and PM DEG lists were then compared to determine shared and unique genes, assess directional agreement (same directionality, up–up, down–down; opposite directionality, up–down, down–up), and compute the Spearman correlation (*r*_*S*_) of log₂ fold-change values across common DEGs.

Gene Ontology (GO) enrichment analyses were independently performed for IMG_sustained_ and PM DEG sets using over-representation analysis (ORA) [41, 42] against the MSigDB “C5” *Mus musculus* collections [43, 44], encompassing biological process (BP), cellular component (CC), and molecular function (MF) categories. Enrichment significance was adjusted by the Benjamini–Hochberg method [45], and results were summarized as *p*_adj_ scores and gene ratios to highlight the most significantly enriched biological pathways in each model.

All analyses were conducted in R Studio (v4.3.1) using the packages *DESeq2*, *clusterProfiler*, *msigdbr*, and *org.Mm.eg.db* for DEG and GO analyses, and *ggplot2*, *ggrepel*, *ggVennDiagram*, and *patchwork* for data visualization.

### Cytokine and NO Assays

Supernatants from IMG cells stimulated with 100 ng/mL LPS or 25 ng/mL IL-4 for 12 or 24 h were collected and analyzed for IL-1β levels using a commercial ELISA kit, following the manufacturer’s instructions. NO production was quantified by measuring nitrite accumulation using the Griess assay, as per the manufacturer’s protocol. Briefly, 150 µL of each sample or standard was mixed with 20 µL of Griess reagent and 130 µL of deionized water in duplicate in a 96-well plate. After incubation for 30 min at room temperature, the absorbance was measured at 548 nm using a Synergy H1 microplate reader (Bio-Tek, Winooski, VT, USA). All ELISA and NO assays were performed in at least three independent experiments.

### Statistical Analysis

The data are presented as mean ± standard error of the mean (SEM) from at least three independent experiments. The qPCR data were analyzed using a mixed-effects model [46] followed by Dunnett’s multiple comparisons test, or one-way analysis of variance (ANOVA) with Dunnett’s post hoc test, as appropriate. The ELISA and NO production data were evaluated using two-way ANOVA followed by Dunnett’s multiple comparisons test. Statistical analyses were performed using GraphPad Prism version 8 (GraphPad Software, La Jolla, CA, USA). A *p* < 0.05 was considered to indicate a statistically significant difference.

## Results

### Dose–Response to Inflammatory and IL-4 Stimuli

Different pro-inflammatory stimuli (LPS, IFNγ, and TNFα) were used in a preliminary screening to determine the best agent and concentration to analyze the pro-inflammatory IMG response. Given that IL-4 has been widely reported to resolve inflammation [11], it was used as the anti-inflammatory agent. A dose–response experiment was performed to select the best concentration. This study was designed to focus on transcriptional changes that remain stable over time. Using the 12–24-h window, we identified a substantial set of genes that stayed consistently deregulated.

#### Pro-inflammatory Stimuli

**Fig. 2:**
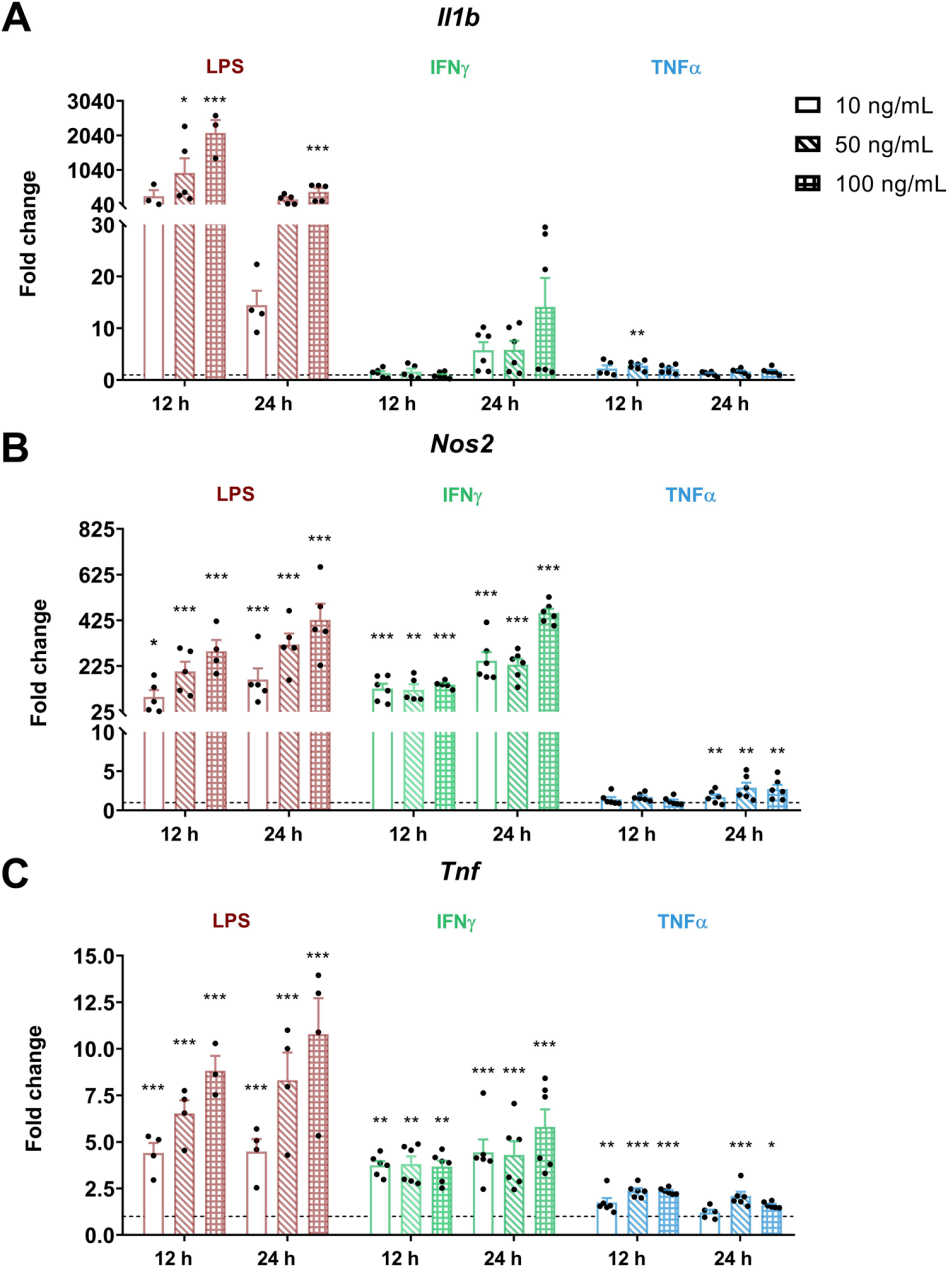
**IMG cell line response to pro-inflammatory stimuli**.Transcript levels of **A** *Il1b*, **B** *Nos2*, and **C** *Tnf* resulting from increasing concentrations (10, 50, or 100 ng/mL) of LPS, IFNγ, or TNFα for 12 h or 24 h. *H3f3a* was used as a reference gene to normalize the fold changes (2^–ΔΔCt^) relative to the control conditions (dotted line). Data are shown as mean ± SEM (*n* ≥ 3). Statistical significance was determined using the mixed-effects model followed by Dunnett’s multiple comparisons test. *p < 0.05, **p < 0.01, ***p < 0.001 vs. control

To characterize the inflammatory phenotype, IMG cells were treated with increasing concentrations (10, 50, and 100 ng/mL) of LPS, IFNγ, and TNFα. The expression of hallmark inflammatory genes (*Il1b*, *Nos2*, and *Tnf)* was assessed by qPCR at 12 and 24 h post-stimulation. Figure 2 shows transcript fold changes for each condition.

All inflammatory stimuli showed a tendency to increase the expression of *Il1b*, *Nos2*, and *Tnf* in IMG cells at both 12 and 24 h, but only LPS elicited a significant response for all genes and at all time points, which, in addition, was the strongest when compared with the other pro-inflammatory agents tested here. This effect was particularly noticeable at the highest concentration used (100 ng/mL), which consistently induced the most robust upregulation, especially in *Il1b*, where the change exceeded 2000-fold at 12 h.

#### IL-4 Stimulation

**Fig. 3:**
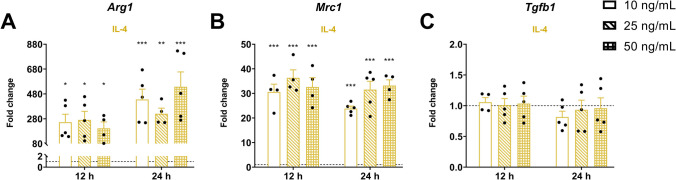
**IMG cell line response to IL-4 stimuli**. Transcript levels of **A** *Arg1*, **B** *Mrc1*, and **C** *Tgfb1* resulting from increasing concentrations (10, 25, or 50 ng/mL) of IL-4 for 12 h or 24 h. *H3f3a* was used as a reference gene to normalize the fold changes (2^–ΔΔCt^) relative to the control conditions (dotted line). Data are shown as mean ± SEM (*n* ≥ 4). Statistical significance was determined using the mixed-effects model followed by Dunnett’s multiple comparisons test. *p < 0.05, **p < 0.01, ***p < 0.001 vs. control

To determine the optimal IL-4 concentration for stimulating IMG cells, a dose–response study was performed using 10, 25, or 50 ng/mL IL-4 for 12 or 24 h. The expression of hallmark anti-inflammatory genes (*Arg1*, *Mrc1*, and *Tgfb1)* was then assessed by qPCR to identify the condition eliciting the strongest response. Figure 3 shows the changes in these genes in response to the different conditions.

*Arg1* was significantly upregulated at all time points and IL-4 concentrations. *Mrc1* expression also increased, but the change was similar at each concentration. In contrast, *Tgfb1* expression was not changed by IL-4 at either time point. Because the 10 and 50 ng/mL IL-4 concentrations did not further enhance the transcriptional response, we selected the 25 ng/mL concentration for the subsequent experiments.

### Selection of Genes with Sustained Expression

Based on the pro-inflammatory screening results, LPS was selected for RNA-seq because it elicited the strongest and most consistent pro-inflammatory transcriptional response across all several timepoints analyzed. After defining the conditions for both pro-inflammatory and anti-inflammatory stimuli, we performed transcriptomic analysis comparing the response of each time point of treatment vs. untreated cells. PCA of variance-stabilized (VST) gene-level counts is shown in Supplementary Information 1. From all the differentially expressed genes at 12 and 24 h for IL-4 and LPS, we focused only on genes whose changes were sustained at both time points (hereafter referred to as “sustained expression”). 

**Fig. 4:**
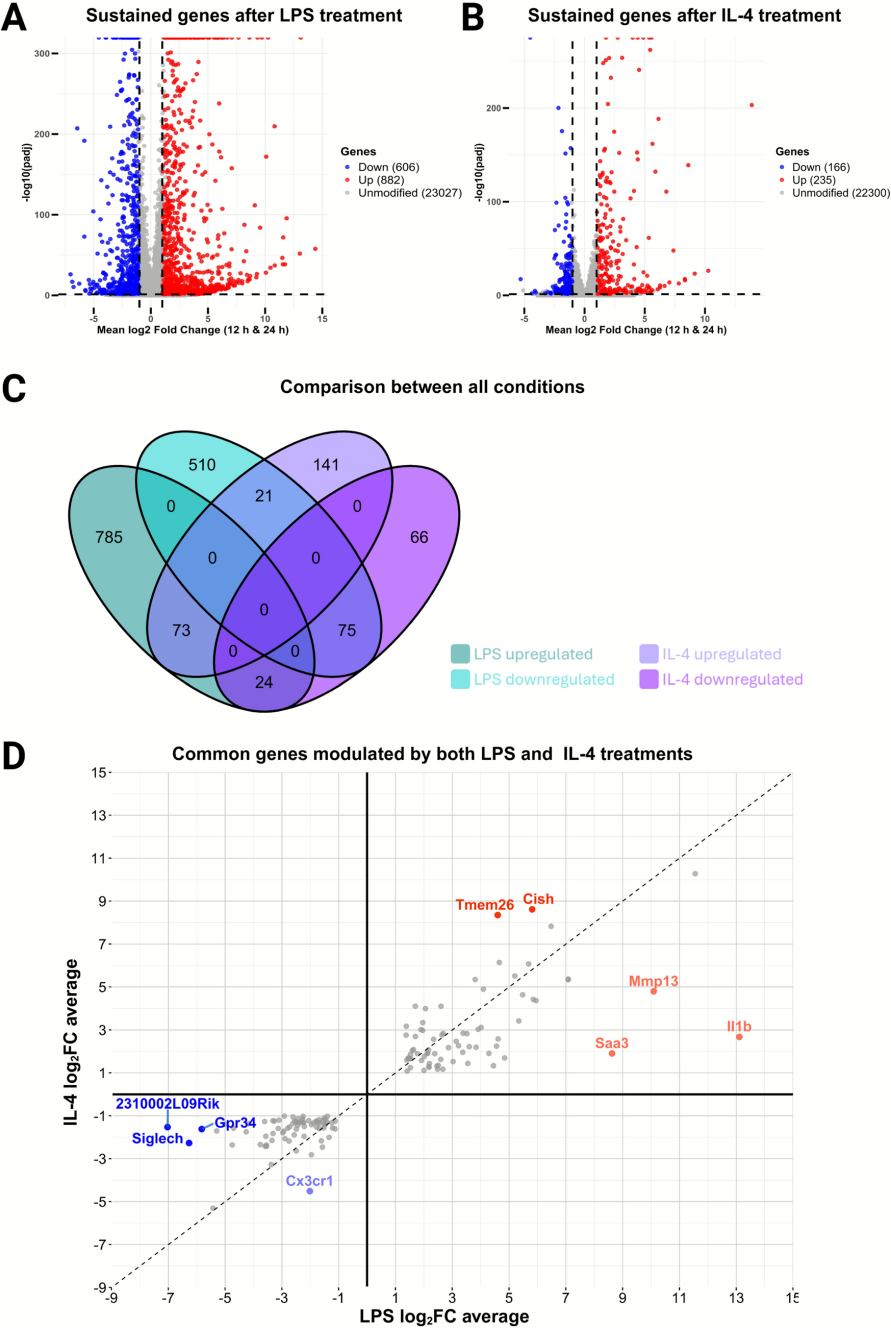
**Transcriptomic analysis of IMG cells following LPS and IL-4 treatments**. **A**, **B** Volcano plots showing sustained differentially expressed genes (DEGs) after 12 h and 24 h of incubation with **A** 100 ng/mL LPS and **B** 25 ng/mL IL-4. DEGs were filtered by log_2_ fold change (log_2_FC) > 1 or < –1 with *p*_adj_ < 0.05. **C** Venn diagram illustrating the overlap of upregulated and downregulated genes across all experimental conditions. Numbers indicate total DEGs, with overlaps representing genes consistently upregulated or downregulated in both treatments. **D** Scatter plot of the average log_2_ fold change of genes commonly modulated by both LPS and IL-4 treatments. Labeled genes highlight those with the most pronounced differential modulation between the two stimuli

There were 606 downregulated and 882 upregulated, genes for the 12LPS + 24LPS conditions (Fig. 4A), vs. 166 downregulated and 235 upregulated genes for the 12IL4 + 24IL4 conditions (Fig. 4B). The Venn diagram (Fig. 4C) and Supplementary Information 2 show 610 downregulated genes and 886 upregulated genes in the LPS conditions, from which we did not consider 3 genes (*F5*, *Gpr137c*, and *Gm15764*) as they were downregulated in 12LPS and upregulated in 24LPS. In addition, we did not consider 1 gene (*Fabp3*) that was upregulated in 12LPS and downregulated in 24LPS. The diagram also shows the 166 downregulated genes and 235 upregulated genes for the IL-4 conditions. We identified 73 common upregulated and 75 common downregulated genes by IL-4 (12IL4 + 24IL4) and LPS (12LPS + 24LPS). *Fabp3* was downregulated by IL-4, but as mentioned above, exhibited different behavior for 12LPS and 24LPS, so it cannot be considered a common downregulated gene for IL-4 and LPS. Finally, 24 genes were downregulated in the 12IL4 and 24IL4 conditions and upregulated in the 12LPS and 24LPS conditions. Moreover, 21 genes were upregulated in both IL-4 conditions and downregulated in both LPS conditions. We called these 45 genes opposite genes because they showed different upregulation in response to LPS and IL-4.

A detailed analysis of the commonly maintained genes (Supplementary Information 2) reveals that some of them can appear in both conditions with a different fold change, which can mask their nonspecific response. Figure 4D shows a scatterplot in which genes with stronger differences are labeled. Some genes, such as *Il1b* or *Mmp13*, appear strongly upregulated with LPS incubation and weakly transcriptionally elevated with IL-4 incubation. The opposite occurs for some genes—for example, *Cx3cr1*.

### Specific Sustained Transcriptomic Signatures for LPS and IL-4

**Fig. 5:**
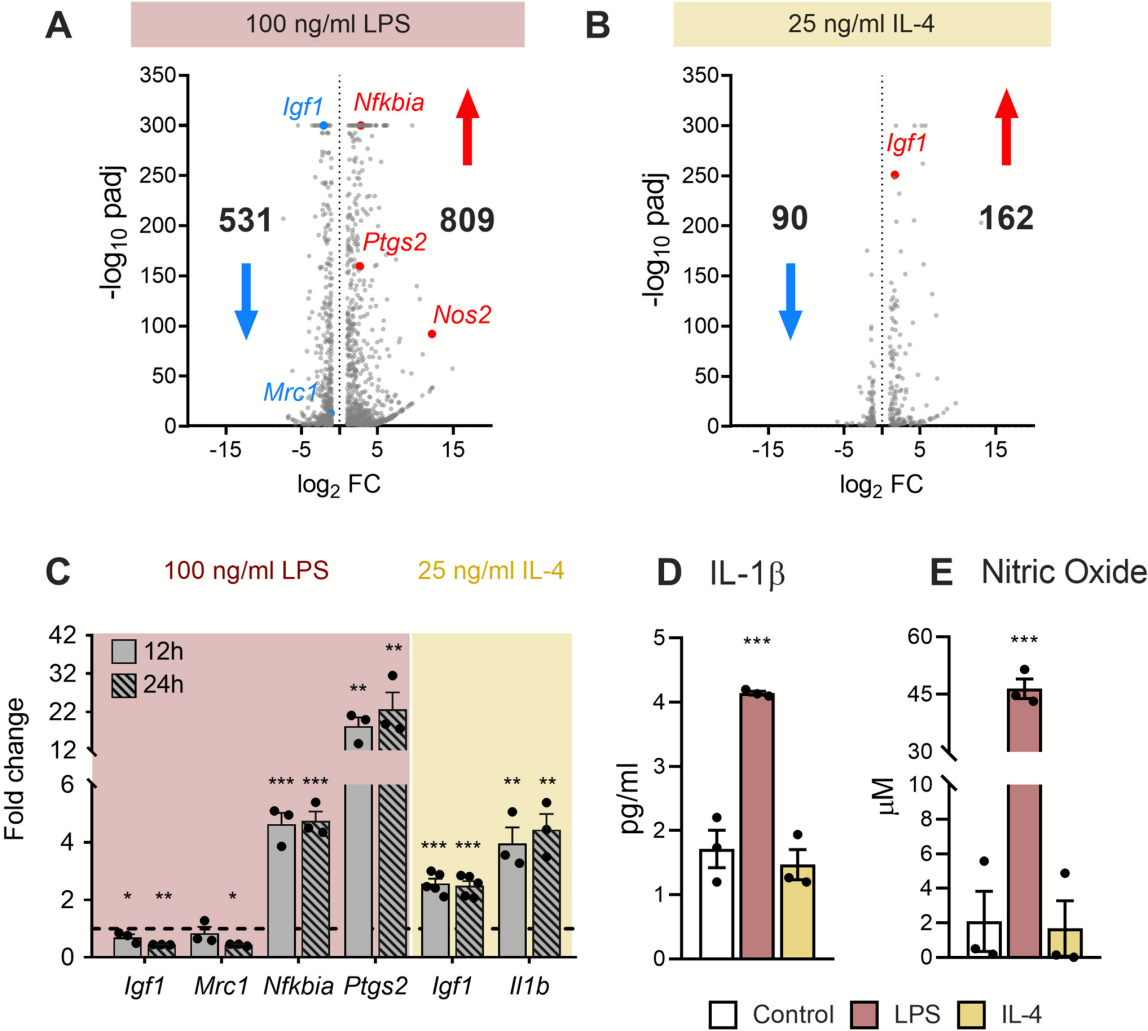
**Transcriptomic profiles of LPS and IL-4-specific responders and RNA-seq validation results**. **A**, **B** Volcano plots showing specific sustained DEGs after **A** 100 ng/mL LPS and **B** 25 ng/mL IL-4 treatments, excluding common responder genes. Downregulated genes are shown in blue and upregulated genes in red. Specific responder genes used for validation are labeled. **C** qPCR validation of RNA-seq data. Transcript levels of *Igf1*, *Mrc1, Nfkbia*, and *Ptgs2* after 100 ng/mL LPS, and *Igf1* and *Il1b* after 25 ng/mL IL-4 were measured at 12 h and 24 h. *H3f3a* was used as a reference gene to normalize the fold changes (2^–ΔΔCt^) relative to the control conditions (dotted line). Data are shown as mean ± SEM (*n* ≥ 3). Statistical significance was determined using the mixed-effects model followed by Dunnett’s multiple comparisons test. *p < 0.05, **p < 0.01, ***p < 0.001 vs. control. **D**, **E** Levels of (D) IL-1β and **E** NO in culture supernatants after 24 h of treatment, measured using the Griess assay and ELISA, respectively. Data are presented as mean ± SEM (*n* ≥ 3). Statistical significance was determined by two-way ANOVA followed by Dunnett’s multiple comparisons test. ***p < 0.001 vs. control

From all genes whose expression was sustainably upregulated (882) or downregulated (606) during LPS incubation, we found 809 upregulated and 531 downregulated genes specifically responding to LPS (genes whose expression is not altered or shows an opposite response under IL-4 stimulation) (Fig. 5A and Supplementary Information 2). Similarly, from the 235 sustainably upregulated and 166 downregulated genes under IL-4 conditions, we identified 162 (up) and 90 (down) as IL-4-specific (Fig. 5B and Supplementary Information 2).

As a corollary, we identified four gene sets: **(**1) LPS-responder genes, (2) IL-4-responder genes, (3) common LPS- and IL-4-responder genes, and (4) genes with opposite regulation by LPS and IL-4. These groups are shown in Supplementary Information 2.

### Validation of the Differentially Expressed Genes (DEGs) Identified by RNA-seq

Since the dose–response experiment and RNA sequencing showed consistent transcript levels for the hallmark genes, we selected additional genes with intermediate expression levels revealed by our RNA-seq analysis for further validation of the transcriptomic profiles by RT-qPCR (Fig. 5A, B).

For LPS stimulation, we examined *Nfkbia*, *Ptgs2*, *Igf1*, and *Mrc1*. RT-qPCR produced fold changes and significant differences that were comparable to those observed with RNA sequencing (Fig. 5C). These results confirm both the induction of pro-inflammatory markers and the downregulation of anti-inflammatory transcripts after LPS treatment. For IL-4 stimulation, we examined *Igf1* and *Il1b*, the latter being a common responder gene for both LPS and IL-4 according to our RNA sequencing data; RT-qPCR and RNA sequencing produced consistent expression patterns (Fig. 5C). Interestingly, when examining the secretion in the culture medium of *Il1b* product, IL-1β, it increased in the supernatant of IMG cells following stimulation with 100 ng/mL LPS, consistent with its transcriptional upregulation, but IL-4 had little to no effect on IL-1β protein in the supernatant, despite its mRNA upregulation (Fig. 5D).

Finally, we examined the NO concentration in the supernatant after the increased mRNA expression of *Nos2* in LPS, but not in IL-4-stimulated cells (Fig. 5A), and consistently, it appears 20-fold higher in LPS-treated cells compared to both untreated and IL-4-treated cells (Fig. 5E).

### GO Signatures for LPS and IL-4-Stimulated IMG

Once the four responder categories were defined, we performed GO enrichment analyses on the LPS- and IL-4–associated DEG sets (LPSup/LPSdown and IL4up/IL4down), excluding the common responders to focus on pathways that differentiate the two stimuli. Of note, the opposite responder genes were included in these GO inputs and were allocated according to directionality (e.g., genes upregulated by LPS and downregulated by IL-4 contribute to the LPSup and IL4down lists, and vice versa). This analysis revealed distinct transcriptomic signatures associated with each stimulus.

**Fig. 6:**
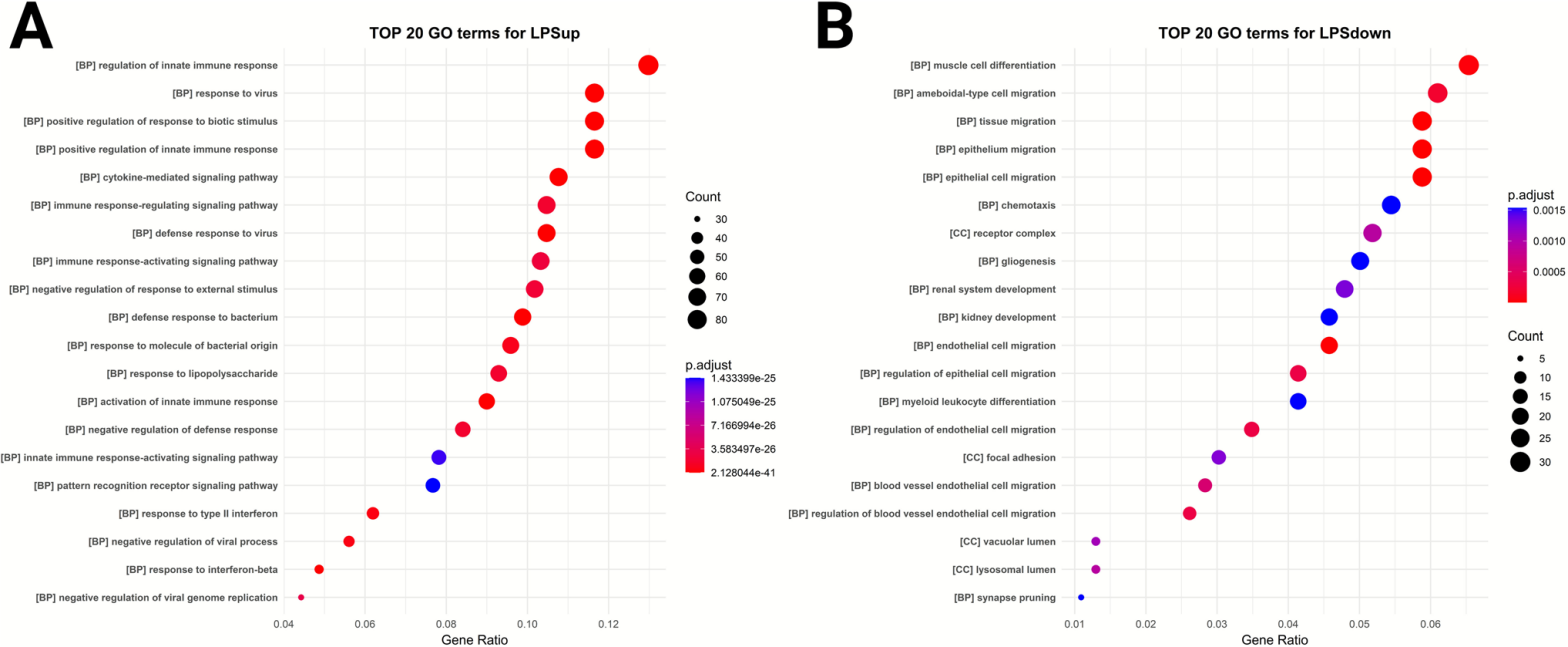
**Gene Ontology (GO) enrichment analysis of differentially expressed genes in IMG cells following LPS stimulation**. **A** Top 20 GO terms for upregulated genes (LPSup) and **B** top 20 GO terms for downregulated genes (LPSdown) after 12–24 h of exposure to 100 ng/mL LPS. The *y*-axis displays the most significantly enriched GO annotations, with GO term processes indicated in brackets. The *x*-axis represents the gene ratio, calculated as the number of differentially expressed genes (DEGs) associated with a GO term divided by the total number of DEGs. Point size reflects the number of genes linked to each term, while point color corresponds to the adjusted *p*-value

There were 1139 significant pathways associated with the LPS-upregulated genes and 268 significant pathways associated with LPS-downregulated genes (Supplementary Information 3). GO enrichment analysis of LPS-responsive genes (Fig. 6) highlighted a strong innate-immune antiviral signature among upregulated transcripts. The top enriched biological processes for LPS-upregulated genes included “response to virus,” “defense response to virus,” and multiple terms related to the “regulation/positive regulation of innate immune response” and “positive regulation of response to biotic stimulus” (Fig. 6A). In contrast, LPS-downregulated genes were enriched for processes related to cell motility and structural dynamics, including “epithelial cell/epithelium migration,” “tissue migration,” “endothelial cell migration,” and “muscle cell differentiation” (Fig. 6B).

**Fig. 7:**
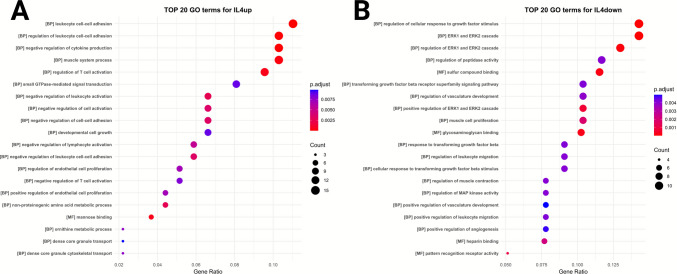
**Gene Ontology (GO) enrichment analysis of differentially expressed genes in IMG cells following IL-4 stimulation**. **A** Top 20 GO terms for upregulated genes (IL4up) and **B** top 20 GO terms for downregulated genes (IL4down) after 12–24 h of exposure to 25 ng/mL IL-4. The *y*-axis displays the most significantly enriched GO annotations, with GO term processes indicated in brackets. The *x*-axis represents the gene ratio, calculated as the number of differentially expressed genes (DEGs) associated with a GO term divided by the total number of DEGs. Point size reflects the number of genes linked to each term, while point color corresponds to the adjusted *p*-value

Similarly, IL-4 upregulated genes were enriched in 186 significant pathways, whereas IL-4 downregulated genes were associated with 236 pathways. GO enrichment analysis of IL-4-responsive genes (Fig. 7) revealed a distinct functional profile compared with LPS. For IL-4-upregulated genes, the most enriched biological processes included “negative regulation of cytokine production,” “leukocyte cell–cell adhesion” (and its regulation), “regulation of T cell activation,” and “muscle system process” (Fig. 7A). Conversely, IL-4-downregulated genes were enriched for signaling and binding-related terms, including “regulation of cellular response to growth factor stimulus,” the “ERK1/ERK2 cascade” (and its regulation), and “sulfur compound binding and glycosaminoglycan binding” (Fig. 7B).

### Comparative Transcriptomic Analysis Between IMG and Primary Microglia

**Fig. 8:**
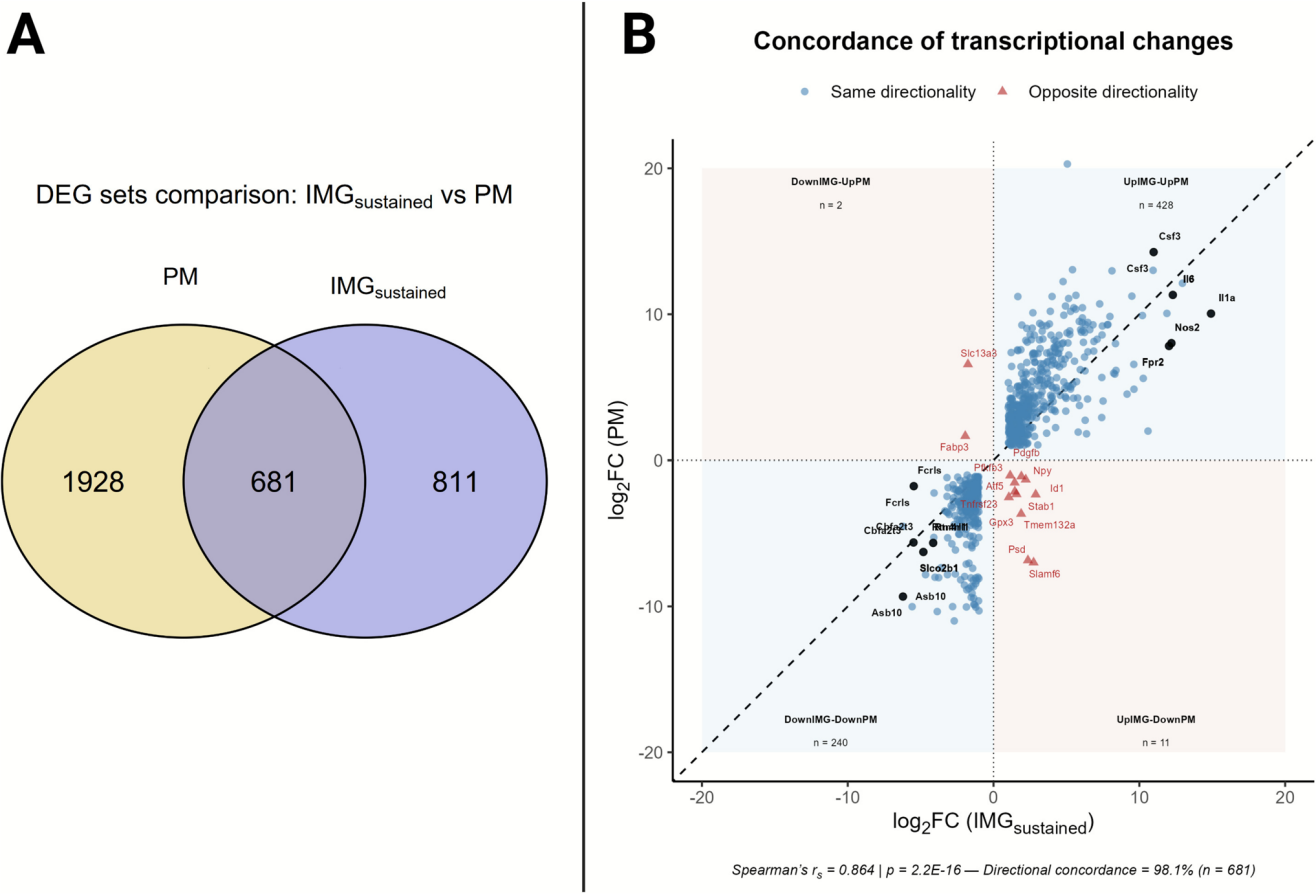
**Concordance in transcriptional directionality and overlap of differentially expressed genes (DEGs)**. **A** Venn diagram showing the overlap between DEG sets from our immortalized microglial line under sustained stimulation (IMG_sustained_) and primary microglia (PM; data from Sabogal-Guáqueta et al., [39]). The applied threshold (*p*_adj_ < 0.05 and |log₂FC|> 1) revealed 681 significant genes shared between both datasets. **B** Scatterplot comparing log₂ fold changes (log₂FC) of significant DEGs between PM and our IMG_sustained_ (threshold, *p*_adj_ < 0.05 and |log₂FC|> 1). Genes showing the same direction of regulation (same directionality) are shown as blue circles, while those with opposite directionality are shown as red triangles. The number of genes in each quadrant is indicated (*n*)*.* Black labels indicate genes with top log₂FC in the IMG_sustained_ dataset. *PM data source: Sabogal-Guáqueta *et al*.* [39]* (GEO series accession number: GSE221013)*

Since we only aimed to characterize the expression of IMG_sustained_ genes, a lower number of DEGs than in the full 24-h dataset of PM was observed (1102 DEGs in IMG_sustained_ vs. 2609 in PM). The overlap between both DEG sets is illustrated in the Venn diagram (Fig. 8A), showing 681 shared genes despite this restriction. The direction of regulation (up- or downregulation) was evaluated in the overlapping genes for testing the concordance between IMG_sustained_ and PM models, which showed highly consistent regulation. Thus, the direction and magnitude of changes were strongly correlated (Spearman’s *r*_*S*_ = 0.864, *p* = 2.2 × 10⁻^16^), and 98.1% of the shared DEGs exhibited concordant directionality. Most common genes clustered within the IMG up—PM up (*n* = 428) and IMG down—PM down (*n* = 240) are shown in Fig. 8B (left down and right-up quadrants). In contrast, very few opposite cases were observed in IMG up—PM down (*n* = 11) and IMG down—PM down (*n* = 2) (Fig. 8B, left up and right down quadrants).

**Fig. 9:**
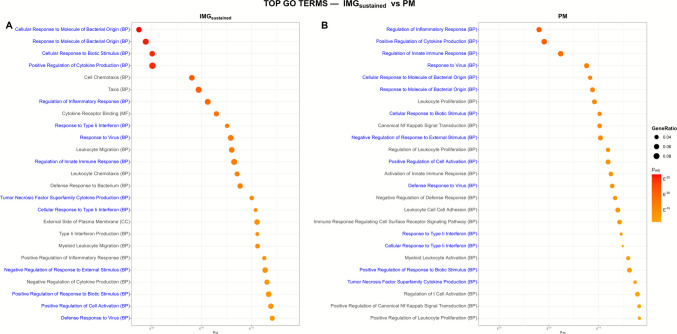
**Comparative analysis of enriched GO terms between IMG**_**sustained**_ **and PM**. Dot plots showing the top 25 enriched Gene Ontology (GO) terms for **A** our immortalized microglial line under sustained stimulation (IMG_sustained_) and **B** primary microglia (PM), ranked by statistical significance (*p*_*ajd*_). Dot size represents the gene ratio (number of DEGs annotated to each term divided by the total number of DEGs in the set), and color indicates enrichment significance (*p*_adj_). GO terms shared by both models are highlighted in blue. *PM data source: Sabogal-Guáqueta *et al*.* [39]* (GEO series accession number: GSE221013)*

Independent GO over-representation analyses revealed extensive functional overlap between IMG_sustained_ and PM GO terms. Thirty-one of the top 50 enriched GO terms were common to both models and were primarily associated with innate immune and inflammatory processes, including response to bacterial molecules, cytokine production, leukocyte activation, chemotaxis, and type I interferon signaling. For readability, Fig. 9 displays the top 25 terms, whereas the top 50 full list is provided in Supplementary Information 3. Interestingly, despite the enrichment significance values varying between datasets due to the different input sizes, the 31 common GO terms correspond to the biological processes category.

## Discussion

We performed a dose–response experiment using IMG cells to test different concentrations of pro-inflammatory agents and IL-4. This study provided additional support for the suitability of IMG as a useful model of microglia whose transcriptomic profiling helps delineate stimulus-induced transcriptional programs in IMG cells and reveals putative therapeutic targets. Based on the results, we chose 100 ng/mL LPS as an appropriate pro-inflammatory agent and concentration given its robust activation of key genes, namely *Nos2*, *Il1b*, and *Tnf*. These genes have been described as hallmarks in other models of LPS-induced microglial response (usually defined in the literature as M1 pro-inflammatory microglia) [27, 47]. We chose 25 ng/mL IL-4 based on the strong transcriptional activation of *Arg1* and *Mrc1*, hallmarks of the microglia IL-4 transcriptional response (usually defined in the literature as M2a anti-inflammatory neuroprotective microglia) [27, 48].

Therefore, we use these concentrations in RNA sequencing, which revealed that LPS and IL-4 modified the transcriptional state of a high number of genes (including upregulation and downregulation). Our data represent a valuable resource allowing to identify gene sets that can help to define different stimulus-induced transcriptional programs, overcoming the limitations of approaches based exclusively on a small set of canonical markers. Incubation for 12 or 24 h with these agents differentiated genes that peaked at the different incubation times from genes that presented sustained expression at both incubation times. We selected the genes with sustained expression as representative of a maintained activated microglial phenotype.

### Common IL-4- and LPS-Responder Genes

Several genes presented activation or repression by both IL-4 and LPS; they are included in the set of common genes modified by the change from the homeostatic microglial state to states linked to the microglial response to pathological conditions, but they are not intrinsically linked to a specific microglial response.

Interestingly, some of these genes exhibit strong activation by LPS but only weak activation by IL-4 and therefore cannot be considered specific responders. The most relevant of these genes is *Il1b*, a canonical pro-inflammatory cytokine gene commonly associated with inflammatory microglial reactivity [49], although its activation by IL-4 has also been described [50]. Consistently, we found *Il1b* upregulation after IL-4 incubation in IMG cells, but its upregulation did not increase IL-1β protein in the culture medium (in contrast to incubation with LPS), adding an extra layer of complexity. *Mmp13* [51] and *Saa3* [52] have also been described in reactive microglia across inflammatory contexts, although they have been cited less often. Our data indicate that these genes do not respond specifically to LPS; therefore, they should not be considered specific markers of reactive microglia under pro-inflammatory stimuli.

Similarly, genes with a high response to IL-4 and a low response to LPS, such as *Cish*, which encodes the cytokine-inducible SH2-containing protein [53, 54], or *Tmem26*, which encodes transmembrane protein 26 [55–57], cannot be considered specific IL-4 responders.

There are also genes downregulated by both LPS and IL-4. The most representative is *Cx3cr1*, a transmembrane receptor of the chemokine fractalkine [58, 59]. In our study, it was strongly downregulated by IL-4 but weakly downregulated by LPS at both incubation times. This common downregulation indicates that *Cx3cr1* is linked to any microglial activation and could explain why its ligand (CX3CL1) has been reported in beneficial or detrimental activities depending on the microglial activation state [60]. Another gene downregulated by both treatments is *Siglech,* which is expressed by parenchymal microglia from developmental stages to adulthood, and its expression is maintained in activated microglia under injury or inflammatory conditions [61, 62]. There are relevant genes strongly downregulated by LPS and weakly by IL-4, which have not yet been directly associated with the microglial response. Examples include *2310002L09Rik*, which encodes a mitochondrial protein with an unknown function [63], and *Pianp*, which encodes Pilr alpha-associated neural protein [64] and has been described to be downregulated in an amyotrophic lateral sclerosis mouse model [65].

Based on our findings, we hypothesize that common LPS- and IL-4-responder genes mirror transcriptional microglial programs under pathological conditions, but they should not be used to characterize specific responsive phases such as the early inflammatory response or the resolution of the inflammation.

### Non-common Genes with Opposite Responses for LPS and IL-4

Several genes exhibited upregulation by LPS and downregulation by IL-4, or vice versa, and define a gene set with opposite transcriptional responses to pro-inflammatory vs. anti-inflammatory stimuli.

Some of the top genes upregulated by LPS and downregulated by IL-4 have been previously associated with microglial functions. Some examples include *Hp*, linked to the free iron-dependent oxidative injury [66]; *Pilra*, associated with AD [67]; and *Thbs1*, recently linked to age-related inflammation (inflammaging) [68]—supporting the idea that the upregulation of these genes is associated with chronic inflammatory states. On the other hand, the top IL-4-upregulated and LPS-downregulated genes, also described in microglia, include *Ccl24*, a chemokine reported to promote an anti-inflammatory microglia response in the brain to protect neurons from death [69]; *Mrc1* has also been reported to be upregulated in microglia under tissue-repair–associated responses in vitro and in vivo, but without a clear functional meaning [70]; and *Clec10a*, also reported to be upregulated in microglia in the resolution phase of autoimmune inflammation [71]. The strong upregulation by LPS, accompanied by downregulation by IL-4, suggests that *Thbs1*, *Pilra*, and *Hp* represent putative therapeutic targets whose upregulation should be decreased to fight inflammation. In contrast, the strong upregulation by IL-4, accompanied by downregulation by LPS, indicates that *Ccl24*, *Mrc1* (CD206), and *Clec10a,* upregulated in chronic inflammation, are putative therapeutic targets.

We suggest that the genes reported here that show an opposite response to LPS and IL-4 could act as microglial response switchers. They seem to be highly relevant in the microglial response to inflammation, opening a window to detect critical pathways in the microglial response.

### Specific LPS-Responder Genes with Strong Over- or Under-expression

Several genes were specifically regulated in response to LPS, without significant changes under IL-4 treatment.

#### Upregulated Genes

Genes that respond selectively to LPS are putative markers of a microglial state responsive to pathological conditions, which would elicit an acute pro-inflammatory response, releasing specific pro-inflammatory cytokines. Some of these genes have been reported to induce a pro-inflammatory state in microglia and, in this study, exhibited the highest changes after LPS incubation for 12 and 24 h. These genes encode very different products, including cytokines, such as *Il1a* [72] or *Il6* [73, 74]; chemokines such as *Cxcl3* [75]; and the colony-stimulating factor 3 (*Csf3*) [76]. This group also includes *Nos2* [77, 78], the scavenger receptor of cell surface *Marco* [79, 80], the inflammatory response protein lipocalin-2 (*Lcn2*) [81], and complement factor B (*Cfb*) [82]. Genes encoding long non-coding RNA (lncRNA) such as *U90926* [83] and *Gm14275* [84] have also been linked to inflammation in microglia. Our study shows that all these genes present sustained expression changes at 24 h and form a consistent panel of LPS-responder genes that can be used as hallmarks for inflammatory microglial responses.

#### Downregulated Genes

Genes strongly downregulated by LPS suggest a number of mechanisms that are inactivated in response to injury. Our study supports relevant changes in protease activation, evidenced by decreases in the genes that encode protease inhibitors such as Serpin B1a (*Serpinb1a*) [85, 86] and proteins that modify protein glycosylation, such as *Cmah* [87] or *Galnt9* [88]. Several molecules directly related to transcription control, including co-activators such as *Mn1* [89, 90] and co-repressors such as *Cbfa2t3* (MTG16) [91], also appear to be downregulated. The downregulation of some genes that encode E3 ubiquitin ligases, including *Klhl3* [92] and *Asb10* [93], or molecules that interact with E3 ubiquitin ligases, such as *Kbtbd11* [94], supports that some proteasome-related proteins involved in homeostatic conditions are degraded after LPS treatment. The role of *Bex6* remains uncertain [95], but its downregulation by LPS suggests that it is involved in mechanisms related to homeostatic states. In addition, *Gm42847* is annotated to encode a lncRNA (based on MGI and GENCODe), but there are no references regarding the functions or biological implications of this gene. Our study links the downregulation of all these genes to a change in the microglial homeostatic response after injury.

We hypothesize that specific LPS-responder genes represent stimulus-induced microglial transcriptional programs elicited by pro-inflammatory stimuli and should be considered putative targets for anti-inflammatory therapies. We think that actions or agents to prevent the expression changes in specific LPS-responder genes could help to decrease the pro-inflammatory microglial response(s). Genes responding selectively to LPS should be considered molecular signatures defining pathogen-induced microglial transcriptional programs. The comparison between LPS-responder genes and genes specifically induced by other pro-inflammatory stimuli could help to identify specific inflammatory pathways in microglia.

### Specific IL-4-Responder Genes with Strong Over- or Under-expression

Another different set of sustained genes included those specifically regulated in response to IL-4, without significant changes under LPS treatment.

#### Upregulated Genes

Following IL-4 incubation, *Arg1* showed the strongest upregulation. It is widely described as the top hallmark IL-4-responder gene [96, 97], and some authors have even described an Arg1 + phenotype in vivo [96]. We found that the IMG cells behave consistently with this response to IL-4, which provides additional support for the use of IMG cells as a microglial model. Our study indicates that *Ang2*, *Mgl2*, *Chil3*, and *Mrc1* (CD206) can be considered hallmarks of the IL-4 response, whose upregulation in the anti-inflammatory microglia terminology has previously been reported [96, 98–104]. We found strong and sustained upregulation of genes that, to our knowledge, have not yet been linked to the microglial inflammatory response. Our study supports their role in the early microglial inflammatory response, particularly with the microglial phenotype related to the resolution of inflammation. These genes include *Rnase2a* (*Ear11*), whose product has been described to enhance neutrophil motility and to promote their recruitment to sites of inflammation [105–107]; *Frmpd1*, which encodes the scaffold protein FERM and PDZ domain containing 1, which has been described to interact with activator of G-protein signaling 3 (AGS3) which in turn interacts with G alpha i subunits [108–110]; and *H2-M5*, which encodes a protein belonging to the major histocompatibility complex (MHC) (as annotated in the MGI and GeneCards databases) [105].

#### Downregulated Genes

Incubation with IL-4 induces the downregulation of a number of genes that can be used to confirm the IL-4-responder phenotype. Interestingly, the top marker genes presented stronger downregulation after incubation for 24 h than after 12 h, indicating a time-dependent transcriptional response to IL-4 and providing support to the observed gene response. Some of the genes showing the strongest downregulation have already been linked to microglial functions—for example, *Megf10*, which encodes a protein that is involved in the synapse phagocytosis [111, 112]. However, other downregulated genes have not been related specifically to microglia or the microglial response to IL-4, despite being involved in different pathologies. In this regard, *Sorbs3*, which encodes vinexin, has been related to cytoskeletal organization and signal transduction [113], and its methylation levels have been related to AD susceptibility [114]. Mutations in *Col4a1*, which encodes the type IV collagen a1 chain, are responsible for an autosomal dominant cerebrovascular disease [115]. Finally, *Scn5a* encodes the cardiac sodium channel Nav1.5 [116], and it has been linked to potentially lethal arrhythmias [117].

Specific IL-4-responder genes would mirror a microglial response related to the resolution of the inflammation, and we suggest that they also constitute putative targets in therapies aimed to fight chronic inflammation. Therefore, we consider that actions or agents addressed to enhance the response of specific IL-4-responder genes could be useful to promote the resolution of inflammation.

### GO Enrichment Analysis Reveals Striking Divergent Transcriptional Responses of IMG Cells to LPS and IL-4 Stimulation

Interpretation of GO terms considers the functional responses described in the literature, the number of pathways related to specific functions, and the adjusted *p*-value. We analyzed the top 50 significant terms to interpret the response of the different stimuli. Overall, the LPS-induced upregulated GO profile reflected activation of innate immune and inflammatory programs, consistent with a microglial response characterized by cytokine release, interferon signaling, and antimicrobial/antiviral defense mechanisms [2, 118, 119]. Conversely, the LPS-induced downregulated GO profile suggested reduced engagement of programs associated with cell motility and structural remodeling, together with pathways linked to regenerative or homeostatic functions. This pattern is consistent with a functional shift away from regenerative/homeostatic roles and toward a more inflammatory and potentially cytotoxic microglial state, as reported in neuroinflammation models [120, 121].

In contrast, the IL-4-induced GO profile was overall consistent with tissue-supportive and immunoregulatory functions. Upregulated terms pointed to pathways related to growth and differentiation programs and to processes involved in tissue remodeling and neurovascular support, which align with restorative microglial functions reported in vivo [11, 122]. In parallel, IL-4 downregulated multiple pathways associated with pro-inflammatory cytokine production and pathogen-related responses, supporting the view that IL-4 promotes a reparative state by dampening inflammatory signaling while favoring vascular and metabolic programs [123, 124].

Of note, there was minimal overlap in upregulated or downregulated GO terms between LPS and IL-4, indicating that each stimulus engages largely non-redundant biological programs. Our results agree with the literature, indicating that LPS and IL-4 induce distinct functional priorities in microglia, with LPS enhancing antimicrobial defense at the expense of structural cell dynamics, while IL-4 promotes growth and signaling recalibration, and selective suppression of inflammatory cytokines [119, 125–127]. However, we identified a subset of common responder genes, indicating that certain shared components of the microglial response are triggered under both conditions. Thus, microglia can integrate multiple signals present in their environment and adopt mixed phenotypes that engage signaling pathways downstream of both pro-inflammatory and regulatory stimuli [50, 128].

Currently, microglial “states” are recommended to be interpreted as context-dependent programs defined by molecular and functional features, rather than as fixed categories [26]. Many of the features described in chronic CNS conditions have also been observed in our IMG model. In particular, we identified GO-enriched transcriptional pathways enriched for innate immune and type I interferon/“antiviral-like” signaling, including type I IFN signatures described in Alzheimer’s disease and related neurodegenerative conditions [129] and STING-dependent type I IFN programs reported to shape microglial phenotype during chronic neurodegeneration [130]. In addition, interferon-responsive microglial programs have been discussed in the context of brain aging [131], and conserved microglial signatures have been reported across aging and neurodegenerative conditions [132].

### Transcriptional Similarity Between Sustained IMG and Primary Microglial Responses

To further explore the physiological relevance of our model, we performed an independent analysis of the raw transcriptomic data reported by Sabogal-Guáqueta et al. [39] to compare LPS responses in IMG and LPS-treated PM.

A partial gene-level overlap (681 shared DEGs) was observed between IMG_sustained_ and PM genes after 24 h of treatment, where the shared genes showed highly concordant directionality and effect sizes (Spearman’s *r*_*S*_ = 0.864; 98.1% directional agreement), and pathway-level analyses revealed substantial functional convergence (31/50 top GO terms shared). It is worth noting that the IMG_sustained_ gene set includes only those genes deregulated in the time window of 12–24 h, while the PM gene set includes all genes deregulated at 24 h of LPS treatments. Together, these results indicate that IMG_sustained_ reproduces key elements and pathways of the LPS-responsive transcriptional signature observed in primary microglia within this time window. These data support the reliability of IMG as a microglial in vitro model.

Additional support for the IMG as a valid in vitro microglial model to assess inflammatory pathway engagement comes from the GO analysis, which reveals the activation of pathways related to innate immune and inflammatory responses, including cytokine production, leukocyte migration, and type I interferon signaling in both IMG and PM models. The few different GO terms observed could involve genes with transient expression in this time frame or be related to the heterogeneity of microglia (different microglia sources and culture environments). In this regard, regional differences in the brain microglia [133] or transcriptional changes in the primary microglia as a consequence of the culture procedure have been reported [134].

Overall, these findings support IMG as a reliable in vitro model which presents a 12-h work window where a high number of genes remains deregulated, allowing to investigate the microglial mechanism of action and screen the response under anti-inflammatory agents as a previous step to the in vivo analysis.

In particular, the sustained gene set (mainly the 681 genes overlapping with PM) identified in this model captures a long-lasting transcriptional response that closely mirrors that observed in primary microglia. The results in these two different models emphasize their value for studying persistent or chronic inflammatory programs in vitro. Although IMG cannot fully replace primary microglia, this cell line represents a robust and reproducible model that can be used in combination with, or as a complementary alternative to, primary cultures for mechanistic and especially for screening studies.

### Limitations

One of the limitations of this study is the restricted temporal sampling (12 and 24 h after treatments), which was designed to characterize the maintained component of the response within this window. Accordingly, we defined “sustained” DEGs as genes significantly deregulated at both 12 h and 24 h, to prioritize changes that persist across timepoints, without implying a return to a basal or homeostatic state. Although transient peaks and earlier-emerging responses may also be functionally important, this study is specifically addressed to characterize the sustained genes in this time window. To minimize the interferences inherent to in vivo transcriptomic studies on primary glial cultures (where the limited presence of other glial cell types may interfere with the microglial response), we employed the immortalized IMG microglial line. While this model does not fully reproduce the transcriptional complexity of primary microglia, it provides a controlled and reproducible system that offers valuable insight into the microglial response. Overall, this study is addressed to establish the transcriptional IMG model, and more detailed studies under different perspectives and experimental designs (functional, proteomic, stimulus response, etc.) will be important to refine and extend its applicability.

In addition, the comparative analysis with primary microglia was restricted to LPS treatment, and a complete validation would require transcriptomic profiling at additional timepoints and under other stimulatory or blocking conditions, as well as functional, metabolic, and proteomic analyses, which would involve multiple additional comparative studies. We think that the transcriptomic evidence described here provides strong support for the suitability of the IMG model and provides novel gene sets related to LPS and IL-4-estimulation, which could be interrogated in other cell culture and in vivo models. Despite the overlap observed with primary microglia, the IMG model should be viewed as a complementary and controlled approach to study microglial responses alongside primary microglia. Further comparative studies will be important to clarify to what extent (and at which levels of resolution) IMG cells recapitulate microglial programs observed in vivo. Finally, although the use of previously published references to interpret microglial responses may introduce some bias in the functional annotation of GO terms, it also provides support to the model since we are comparing results from independent laboratories and reduces the use of animals.

## Conclusions

This study presents the first comprehensive transcriptomic comparison of LPS- and IL-4-responsive genes in IMG cells. These data offer a valuable resource for identifying stimulus-based transcriptional signatures that operationally describe microglial response programs under defined in vitro conditions, overcoming the limitations of approaches based on a narrow panel of canonical markers. RNA sequencing under LPS and IL-4 stimulation revealed four sustained gene sets that help characterize distinct response patterns in microglia, including stimulus-specific, common, and opposite responder programs. We hypothesize that the common LPS- and IL-4-responder gene set is indicative of a general response of microglia to pathological conditions, while the LPS- and IL-4-responder gene sets characterize specific microglial responses under pro-inflammatory and anti-inflammatory stimuli, respectively. Finally, the presence of genes that exhibit LPS-induced upregulation and IL-4-induced downregulation, and vice versa, leads us to hypothesize that they act as metabolic switches between certain microglial responses. Moreover, the overlapping of the transcriptional response of IMG cells compared to primary microglia reveals highly similar gene expression and GO term profiles under the same LPS stimulation.

## Supplementary Information

Below is the link to the electronic supplementary material.

ESM 1 (DOCX 112 KB)

ESM 2 (XLSX 371 KB)

ESM 3 (XLSX 197 KB)

## Data Availability

The data that support the findings of this study are available in the supplementary material of this article. The datasets generated through Sabogal-Guáqueta et al. ([39]) and re-analyzed for this study are available through GEO Series accession number GSE221013. The data that support the findings of the IMG cells are available from the corresponding author upon reasonable request.

## References

[1] Arcuri C, Mecca C, Bianchi R et al (2017) The pathophysiological role of microglia in dynamic surveillance, phagocytosis and structural remodeling of the developing CNS. Front Mol Neurosci 10:191. 10.3389/fnmol.2017.00191 28674485 10.3389/fnmol.2017.00191 PMC5474494

[2] Colonna M, Butovsky O (2017) Microglia function in the central nervous system during health and neurodegeneration. Annu Rev Immunol 35:441–468. 10.1146/annurev-immunol-051116-052358 28226226 10.1146/annurev-immunol-051116-052358 PMC8167938

[3] Thion MS, Ginhoux F, Garel S (2018) Microglia and early brain development: an intimate journey. Science 362:185–189. 10.1126/science.aat0474 30309946 10.1126/science.aat0474

[4] Chavez J, Le AA, Quintanilla J et al (2025) Microglia support both the singular form of LTP expressed by the lateral perforant path and episodic memory. J Neurosci 45:e1322242025. 10.1523/JNEUROSCI.1322-24.2025 40404354 10.1523/JNEUROSCI.1322-24.2025 PMC12199551

[5] Schwartz M (2025) Why resident microglial-like cells were missed in the peripheral nervous system. Cell Res. 10.1038/s41422-025-01119-2 40229554 10.1038/s41422-025-01119-2 PMC12589639

[6] Stansley B, Post J, Hensley K (2012) A comparative review of cell culture systems for the study of microglial biology in Alzheimer’s disease. J Neuroinflammation 9:115. 10.1186/1742-2094-9-115 22651808 10.1186/1742-2094-9-115 PMC3407712

[7] Gao C, Jiang J, Tan Y, Chen S (2023) Microglia in neurodegenerative diseases: mechanism and potential therapeutic targets. Signal Transduct Target Ther 8:359. 10.1038/s41392-023-01588-0 37735487 10.1038/s41392-023-01588-0 PMC10514343

[8] Shi F-D, Yong VW (2025) Neuroinflammation across neurological diseases. Science 388:eadx0043. 10.1126/science.adx0043 40536983 10.1126/science.adx0043

[9] Savitz SI, Cox CS (2016) Concise review: cell therapies for stroke and traumatic brain injury: targeting microglia. Stem Cells 34:537–542. 10.1002/stem.2253 26844424 10.1002/stem.2253

[10] Scott MC, Bedi SS, Olson SD et al (2021) Microglia as therapeutic targets after neurological injury: strategy for cell therapy. Expert Opin Ther Targets 25:365–380. 10.1080/14728222.2021.1934447 34029505 10.1080/14728222.2021.1934447 PMC8759629

[11] Orihuela R, McPherson CA, Harry GJ (2016) Microglial M1/M2 polarization and metabolic states. Br J Pharmacol 173:649–665. 10.1111/bph.13139 25800044 10.1111/bph.13139 PMC4742299

[12] He Y, Yao X, Taylor N et al (2018) RNA sequencing analysis reveals quiescent microglia isolation methods from postnatal mouse brains and limitations of BV2 cells. J Neuroinflammation 15:153. 10.1186/s12974-018-1195-4 29788964 10.1186/s12974-018-1195-4 PMC5964710

[13] Horvath RJ, Nutile-McMenemy N, Alkaitis MS, Deleo JA (2008) Differential migration, LPS-induced cytokine, chemokine, and NO expression in immortalized BV-2 and HAPI cell lines and primary microglial cultures. J Neurochem 107:557–569. 10.1111/j.1471-4159.2008.05633.x 18717813 10.1111/j.1471-4159.2008.05633.x PMC2581646

[14] de Jong EK, de Haas AH, Brouwer N et al (2008) Expression of CXCL4 in microglia in vitro and in vivo and its possible signaling through CXCR3. J Neurochem 105:1726–1736. 10.1111/j.1471-4159.2008.05267.x 18248618 10.1111/j.1471-4159.2008.05267.x

[15] Das A, Kim SH, Arifuzzaman S et al (2016) Transcriptome sequencing reveals that LPS-triggered transcriptional responses in established microglia BV2 cell lines are poorly representative of primary microglia. J Neuroinflammation 13:182. 10.1186/s12974-016-0644-1 27400875 10.1186/s12974-016-0644-1 PMC4940985

[16] Henn A, Lund S, Hedtjärn M, et al (2009) The suitability of BV2 cells as alternative model system for primary microglia cultures or for animal experiments examining brain inflammation. ALTEX 26:83–94. 10.14573/altex.2009.2.83 10.14573/altex.2009.2.83 19565166

[17] McCarthy RC, Lu D-Y, Alkhateeb A et al (2016) Characterization of a novel adult murine immortalized microglial cell line and its activation by amyloid-beta. J Neuroinflammation 13:21. 10.1186/s12974-016-0484-z 26819091 10.1186/s12974-016-0484-z PMC4730646

[18] Viil J, Somelar-Duracz K, Jaako K et al (2023) Characterization of IMG microglial cell line as a valuable in vitro tool for NLRP3 inflammasome studies. Cell Mol Neurobiol 43:2053–2069. 10.1007/s10571-022-01285-6 36163404 10.1007/s10571-022-01285-6 PMC11412188

[19] Mantovani A, Sica A, Sozzani S et al (2004) The chemokine system in diverse forms of macrophage activation and polarization. Trends Immunol 25:677–686. 10.1016/j.it.2004.09.015 15530839 10.1016/j.it.2004.09.015

[20] Martinez FO, Gordon S (2014) The M1 and M2 paradigm of macrophage activation: time for reassessment. F1000Prime Rep 6:13. 10.12703/P6-13 24669294 10.12703/P6-13 PMC3944738

[21] Qie S, Ran Y, Lu X et al (2020) Candesartan modulates microglia activation and polarization via NF-κB signaling pathway. Int J Immunopathol Pharmacol 34:2058738420974900. 10.1177/2058738420974900 33237822 10.1177/2058738420974900 PMC7691946

[22] Kisucká A, Bimbová K, Bačová M et al (2021) Activation of neuroprotective microglia and astrocytes at the lesion site and in the adjacent segments is crucial for spontaneous locomotor recovery after spinal cord injury. Cells 10:1943. 10.3390/cells10081943 34440711 10.3390/cells10081943 PMC8394075

[23] Wendimu MY, Hooks SB (2022) Microglia phenotypes in aging and neurodegenerative diseases. Cells 11:2091. 10.3390/cells11132091 35805174 10.3390/cells11132091 PMC9266143

[24] Imamichi T, Yang J, Chen Q et al (2025) Interleukin-27-polarized HIV-resistant M2 macrophages are a novel subtype of macrophages that express distinct antiviral gene profiles in individual cells: implication for the antiviral effect via different mechanisms in the individual cell-dependent manner. Front Immunol 16:1550699. 10.3389/fimmu.2025.1550699 40129989 10.3389/fimmu.2025.1550699 PMC11931227

[25] Devanney NA, Stewart AN, Gensel JC (2020) Microglia and macrophage metabolism in CNS injury and disease: the role of immunometabolism in neurodegeneration and neurotrauma. Exp Neurol 329:113310. 10.1016/j.expneurol.2020.113310 32289316 10.1016/j.expneurol.2020.113310 PMC7237336

[26] Paolicelli RC, Sierra A, Stevens B et al (2022) Microglia states and nomenclature: a field at its crossroads. Neuron 110:3458–3483. 10.1016/j.neuron.2022.10.020 36327895 10.1016/j.neuron.2022.10.020 PMC9999291

[27] Wang J, He W, Zhang J (2023) A richer and more diverse future for microglia phenotypes. Heliyon 9:e14713. 10.1016/j.heliyon.2023.e14713 37025898 10.1016/j.heliyon.2023.e14713 PMC10070543

[28] Taylor S, Wakem M, Dijkman G et al (2010) A practical approach to RT-qPCR-publishing data that conform to the MIQE guidelines. Methods 50:S1-5. 10.1016/j.ymeth.2010.01.005 20215014 10.1016/j.ymeth.2010.01.005

[29] Livak KJ, Schmittgen TD (2001) Analysis of relative gene expression data using real-time quantitative PCR and the 2(-Delta Delta C(T)) method. Methods 25:402–408. 10.1006/meth.2001.1262 11846609 10.1006/meth.2001.1262

[30] Parkhomchuk D, Borodina T, Amstislavskiy V et al (2009) Transcriptome analysis by strand-specific sequencing of complementary DNA. Nucleic Acids Res 37:e123. 10.1093/nar/gkp596 19620212 10.1093/nar/gkp596 PMC2764448

[31] Mortazavi A, Williams BA, McCue K et al (2008) Mapping and quantifying mammalian transcriptomes by RNA-Seq. Nat Methods 5:621–628. 10.1038/nmeth.1226 18516045 10.1038/nmeth.1226 PMC13303166

[32] Pertea M, Pertea GM, Antonescu CM et al (2015) StringTie enables improved reconstruction of a transcriptome from RNA-seq reads. Nat Biotechnol 33:290–295. 10.1038/nbt.3122 25690850 10.1038/nbt.3122 PMC4643835

[33] Liao Y, Smyth GK, Shi W (2014) FeatureCounts: an efficient general purpose program for assigning sequence reads to genomic features. Bioinformatics 30:923–930. 10.1093/bioinformatics/btt656 24227677 10.1093/bioinformatics/btt656

[34] Anders S, Huber W (2010) Differential expression analysis for sequence count data. Genome Biol 11:R106. 10.1186/gb-2010-11-10-r106 20979621 10.1186/gb-2010-11-10-r106 PMC3218662

[35] Yan (颜林林) L (2025) yanlinlin82/ggvenn

[36] Yu G, Wang L-G, Han Y, He Q-Y (2012) ClusterProfiler: an R package for comparing biological themes among gene clusters. OMICS 16:284–287. 10.1089/omi.2011.0118 22455463 10.1089/omi.2011.0118 PMC3339379

[37] Pagès RNAseqData.HNRNPC.bam.chr14. In: Bioconductor. http://bioconductor.org/packages/RNAseqData.HNRNPC.bam.chr14/. Accessed 4 Nov 2025

[38] Sepulveda JL (2020) Using R and bioconductor in clinical genomics and transcriptomics. J Mol Diagn 22:3–20. 10.1016/j.jmoldx.2019.08.006 31605800 10.1016/j.jmoldx.2019.08.006

[39] Sabogal-Guáqueta AM, Marmolejo-Garza A, Trombetta-Lima M et al (2023) Species-specific metabolic reprogramming in human and mouse microglia during inflammatory pathway induction. Nat Commun 14:6454. 10.1038/s41467-023-42096-7 37833292 10.1038/s41467-023-42096-7 PMC10575978

[40] Love MI, Huber W, Anders S (2014) Moderated estimation of fold change and dispersion for RNA-seq data with DESeq2. Genome Biol 15:550. 10.1186/s13059-014-0550-8 25516281 10.1186/s13059-014-0550-8 PMC4302049

[41] Khatri P, Sirota M, Butte AJ (2012) Ten years of pathway analysis: current approaches and outstanding challenges. PLoS Comput Biol 8:e1002375. 10.1371/journal.pcbi.1002375 22383865 10.1371/journal.pcbi.1002375 PMC3285573

[42] Wu T, Hu E, Xu S et al (2021) ClusterProfiler 4.0: a universal enrichment tool for interpreting omics data. Innovation 2:100141. 10.1016/j.xinn.2021.100141 34557778 10.1016/j.xinn.2021.100141 PMC8454663

[43] Liberzon A, Birger C, Thorvaldsdóttir H et al (2015) The Molecular Signatures Database (MSigDB) hallmark gene set collection. Cell Syst 1:417–425. 10.1016/j.cels.2015.12.004 26771021 10.1016/j.cels.2015.12.004 PMC4707969

[44] Gene Ontology Consortium (2021) The gene ontology resource: enriching a GOld mine. Nucleic Acids Res 49:D325–D334. 10.1093/nar/gkaa1113 33290552 10.1093/nar/gkaa1113 PMC7779012

[45] Benjamini Y, Hochberg Y (1995) Controlling the false discovery rate: a practical and powerful approach to multiple testing. J Roy Stat Soc: Ser B (Methodol) 57:289–300

[46] Gomes DGE (2022) Should i use fixed effects or random effects when i have fewer than five levels of a grouping factor in a mixed-effects model? PeerJ 10:e12794. 10.7717/peerj.12794 35116198 10.7717/peerj.12794 PMC8784019

[47] Ji J, Xue T-F, Guo X-D et al (2018) Antagonizing peroxisome proliferator-activated receptor γ facilitates M1-to-M2 shift of microglia by enhancing autophagy via the LKB1-AMPK signaling pathway. Aging Cell 17:e12774. 10.1111/acel.12774 29740932 10.1111/acel.12774 PMC6052482

[48] Kalkman HO, Feuerbach D (2017) Microglia M2A polarization as potential link between food allergy and autism spectrum disorders. Pharmaceuticals (Basel) 10:95. 10.3390/ph10040095 29232822 10.3390/ph10040095 PMC5748650

[49] Akhtar F, Rouse CA, Catano G et al (2017) Acute maternal oxidant exposure causes susceptibility of the fetal brain to inflammation and oxidative stress. J Neuroinflammation 14:195. 10.1186/s12974-017-0965-8 28962577 10.1186/s12974-017-0965-8 PMC5622443

[50] Fenn AM, Hall JCE, Gensel JC et al (2014) IL-4 signaling drives a unique arginase+/IL-1β+ microglia phenotype and recruits macrophages to the inflammatory CNS: consequences of age-related deficits in IL-4Rα after traumatic spinal cord injury. J Neurosci 34:8904–8917. 10.1523/JNEUROSCI.1146-14.2014 24966389 10.1523/JNEUROSCI.1146-14.2014 PMC4069360

[51] Sánchez K, Maguire-Zeiss K (2020) MMP13 expression is increased following mutant α-synuclein exposure and promotes inflammatory responses in microglia. Front Neurosci 14:585544. 10.3389/fnins.2020.585544 33343280 10.3389/fnins.2020.585544 PMC7738560

[52] Feng H, Zhou W, Yang Y et al (2024) Serum amyloid A aggravates endotoxin-induced ocular inflammation through the regulation of retinal microglial activation. FASEB J 38:e23389. 10.1096/fj.202301150RRR 38153347 10.1096/fj.202301150RRR

[53] Yoshimura A, Ohkubo T, Kiguchi T et al (1995) A novel cytokine-inducible gene CIS encodes an SH2-containing protein that binds to tyrosine-phosphorylated interleukin 3 and erythropoietin receptors. EMBO J 14:2816–2826. 10.1002/j.1460-2075.1995.tb07281.x 7796808 10.1002/j.1460-2075.1995.tb07281.x PMC398400

[54] Juknat A, Pietr M, Kozela E et al (2013) Microarray and pathway analysis reveal distinct mechanisms underlying cannabinoid-mediated modulation of LPS-induced activation of BV-2 microglial cells. PLoS ONE 8:e61462. 10.1371/journal.pone.0061462 23637839 10.1371/journal.pone.0061462 PMC3634783

[55] Han G, Zhou S, Shen J et al (2023) The role of TMEM26 in disrupting tight junctions and activating NF-κB signaling to promote epithelial-mesenchymal transition in esophageal squamous cell carcinoma. Clinics 78:100276. 10.1016/j.clinsp.2023.100276 37611445 10.1016/j.clinsp.2023.100276 PMC10466919

[56] Jablonski KA, Amici SA, Webb LM et al (2015) Novel markers to delineate murine M1 and M2 macrophages. PLoS ONE 10:e0145342. 10.1371/journal.pone.0145342 26699615 10.1371/journal.pone.0145342 PMC4689374

[57] Vitale I, Manic G, Coussens LM et al (2019) Macrophages and metabolism in the tumor microenvironment. Cell Metab 30:36–50. 10.1016/j.cmet.2019.06.001 31269428 10.1016/j.cmet.2019.06.001

[58] Chapman GA, Moores K, Harrison D et al (2000) Fractalkine cleavage from neuronal membranes represents an acute event in the inflammatory response to excitotoxic brain damage. J Neurosci 20:RC87. 10.1523/JNEUROSCI.20-15-j0004.2000 10899174 10.1523/JNEUROSCI.20-15-j0004.2000 PMC6772533

[59] Fuhrmann M, Bittner T, Jung CKE et al (2010) Microglial Cx3cr1 knockout prevents neuron loss in a mouse model of Alzheimer’s disease. Nat Neurosci 13:411–413. 10.1038/nn.2511 20305648 10.1038/nn.2511 PMC4072212

[60] Lauro C, Catalano M, Trettel F, Limatola C (2015) Fractalkine in the nervous system: neuroprotective or neurotoxic molecule? Ann N Y Acad Sci 1351:141–148. 10.1111/nyas.12805 26084002 10.1111/nyas.12805

[61] Konishi H, Kobayashi M, Kunisawa T et al (2017) Siglec-H is a microglia-specific marker that discriminates microglia from CNS-associated macrophages and CNS-infiltrating monocytes. Glia 65:1927–1943. 10.1002/glia.23204 28836308 10.1002/glia.23204

[62] Bakina O, Conrad T, Mitic N et al (2024) In situ patch-seq analysis of microglia reveals a lack of stress genes as found in FACS-isolated microglia. PLoS ONE 19:e0302376. 10.1371/journal.pone.0302376 38990806 10.1371/journal.pone.0302376 PMC11239014

[63] Pagliarini DJ, Calvo SE, Chang B et al (2008) A mitochondrial protein compendium elucidates complex I disease biology. Cell 134:112–123. 10.1016/j.cell.2008.06.016 18614015 10.1016/j.cell.2008.06.016 PMC2778844

[64] Biswas S, Adrian M, Evdokimov K et al (2015) Counter-regulation of the ligand-receptor pair Leda-1/Pianp and Pilrα during the LPS-mediated immune response of murine macrophages. Biochem Biophys Res Commun 464:1078–1083. 10.1016/j.bbrc.2015.07.079 26188512 10.1016/j.bbrc.2015.07.079

[65] Hunter M, Spiller KJ, Dominique MA et al (2021) Microglial transcriptome analysis in the rNLS8 mouse model of TDP-43 proteinopathy reveals discrete expression profiles associated with neurodegenerative progression and recovery. Acta Neuropathol Commun 9:140. 10.1186/s40478-021-01239-x 34412701 10.1186/s40478-021-01239-x PMC8377972

[66] Morimoto M, Nakano T, Egashira S et al (2022) Haptoglobin regulates macrophage/microglia-induced inflammation and prevents ischemic brain damage via binding to HMGB1. J Am Heart Assoc 11:e024424. 10.1161/JAHA.121.024424 35243897 10.1161/JAHA.121.024424 PMC9075294

[67] Smith AM, Davey K, Tsartsalis S et al (2022) Diverse human astrocyte and microglial transcriptional responses to Alzheimer’s pathology. Acta Neuropathol 143:75–91. 10.1007/s00401-021-02372-6 34767070 10.1007/s00401-021-02372-6 PMC8732962

[68] Ramalingam P, Gutkin MC, Poulos MG et al (2025) Suppression of thrombospondin-1-mediated inflammaging prolongs hematopoietic health span. Sci Immunol 10:eads1556. 10.1126/sciimmunol.ads1556 39752538 10.1126/sciimmunol.ads1556 PMC12068530

[69] Xu Y, Li Y, Wang C et al (2023) The reciprocal interactions between microglia and T cells in Parkinson’s disease: a double-edged sword. J Neuroinflammation 20:33. 10.1186/s12974-023-02723-y 36774485 10.1186/s12974-023-02723-y PMC9922470

[70] Hu X, Li P, Guo Y et al (2012) Microglia/macrophage polarization dynamics reveal novel mechanism of injury expansion after focal cerebral ischemia. Stroke 43:3063–3070. 10.1161/STROKEAHA.112.659656 22933588 10.1161/STROKEAHA.112.659656

[71] Ilarregui JM, Kooij G, Rodríguez E et al (2019) Macrophage galactose-type lectin (MGL) is induced on M2 microglia and participates in the resolution phase of autoimmune neuroinflammation. J Neuroinflammation 16:130. 10.1186/s12974-019-1522-4 31248427 10.1186/s12974-019-1522-4 PMC6598247

[72] Di Paolo NC, Shayakhmetov DM (2016) Interleukin 1α and the inflammatory process. Nat Immunol 17:906–913. 10.1038/ni.3503 27434011 10.1038/ni.3503 PMC5152572

[73] Tanaka T, Narazaki M, Kishimoto T (2014) IL-6 in inflammation, immunity, and disease. Cold Spring Harb Perspect Biol 6:a016295. 10.1101/cshperspect.a016295 25190079 10.1101/cshperspect.a016295 PMC4176007

[74] Kaur S, Bansal Y, Kumar R, Bansal G (2020) A panoramic review of IL-6: structure, pathophysiological roles and inhibitors. Bioorg Med Chem 28:115327. 10.1016/j.bmc.2020.115327 31992476 10.1016/j.bmc.2020.115327

[75] Qu X, Dou B, Yang R et al (2023) C-X-C motif chemokine 3 promotes the inflammatory response of microglia after *Escherichia coli*-induced meningitis. Int J Mol Sci 24:10432. 10.3390/ijms241310432 37445610 10.3390/ijms241310432 PMC10341832

[76] Biundo F, Chitu V, Tindi J et al (2023) Elevated granulocyte colony stimulating factor (CSF) causes cerebellar deficits and anxiety in a model of CSF-1 receptor related leukodystrophy. Glia 71:775–794. 10.1002/glia.24310 36433736 10.1002/glia.24310 PMC9868112

[77] Rodríguez-Gómez JA, Kavanagh E, Engskog-Vlachos P et al (2020) Microglia: agents of the CNS pro-inflammatory response. Cells 9:1717. 10.3390/cells9071717 32709045 10.3390/cells9071717 PMC7407646

[78] Gottschalk G, Peterson D, Knox K et al (2022) Elevated ATG13 in serum of patients with ME/CFS stimulates oxidative stress response in microglial cells via activation of receptor for advanced glycation end products (RAGE). Mol Cell Neurosci 120:103731. 10.1016/j.mcn.2022.103731 35487443 10.1016/j.mcn.2022.103731

[79] Granucci F, Petralia F, Urbano M et al (2003) The scavenger receptor MARCO mediates cytoskeleton rearrangements in dendritic cells and microglia. Blood 102:2940–2947. 10.1182/blood-2002-12-3651 12842997 10.1182/blood-2002-12-3651

[80] Han RT, Vainchtein ID, Schlachetzki JCM et al (2023) Microglial pattern recognition via IL-33 promotes synaptic refinement in developing corticothalamic circuits in mice. J Exp Med 220:e20220605. 10.1084/jem.20220605 36520518 10.1084/jem.20220605 PMC9757845

[81] Afridi R, Kim J-H, Bhusal A et al (2024) Lipocalin-2 as a mediator of neuroimmune communication. J Leukoc Biol 116:357–368. 10.1093/jleuko/qiad157 38149462 10.1093/jleuko/qiad157

[82] Sarma JV, Ward PA (2011) The complement system. Cell Tissue Res 343:227–235. 10.1007/s00441-010-1034-0 20838815 10.1007/s00441-010-1034-0 PMC3097465

[83] Chen J, Jin J, Zhang X et al (2021) Microglial lnc-U90926 facilitates neutrophil infiltration in ischemic stroke via MDH2/CXCL2 axis. Mol Ther 29:2873–2885. 10.1016/j.ymthe.2021.04.025 33895326 10.1016/j.ymthe.2021.04.025 PMC8417913

[84] Babagana M, Oh K-S, Chakraborty S et al (2021) Hedgehog dysregulation contributes to tissue-specific inflammaging of resident macrophages. Aging 13:19207–19229. 10.18632/aging.203422 34390567 10.18632/aging.203422 PMC8386529

[85] Choi YJ, Kim S, Choi Y et al (2019) SERPINB1-mediated checkpoint of inflammatory caspase activation. Nat Immunol 20:276–287. 10.1038/s41590-018-0303-z 30692621 10.1038/s41590-018-0303-z PMC6450391

[86] Ye G, Wang Z, Chen P et al (2025) Serpina3n in neonatal microglia mediates its protective role for damaged adult microglia by alleviating extracellular matrix remodeling-induced tunneling nanotubes degradation in a cell model of traumatic brain injury. Neuroscience 565:1–9. 10.1016/j.neuroscience.2024.11.066 39613247 10.1016/j.neuroscience.2024.11.066

[87] Shemer A, Grozovski J, Tay TL et al (2018) Engrafted parenchymal brain macrophages differ from microglia in transcriptome, chromatin landscape and response to challenge. Nat Commun 9:5206. 10.1038/s41467-018-07548-5 30523248 10.1038/s41467-018-07548-5 PMC6284018

[88] Peng Y, Liu J, Sun L et al (2024) GALNT9 enrichment attenuates MPP+-induced cytotoxicity by ameliorating protein aggregations containing α-synuclein and mitochondrial dysfunction. Biol Direct 19:77. 10.1186/s13062-024-00524-8 39237967 10.1186/s13062-024-00524-8 PMC11378468

[89] van Wely KHM, Molijn AC, Buijs A et al (2003) The MN1 oncoprotein synergizes with coactivators RAC3 and p300 in RAR-RXR-mediated transcription. Oncogene 22:699–709. 10.1038/sj.onc.1206124 12569362 10.1038/sj.onc.1206124

[90] Meester-Smoor MA, Janssen MJFW, Grosveld GC et al (2008) MN1 affects expression of genes involved in hematopoiesis and can enhance as well as inhibit RAR/RXR-induced gene expression. Carcinogenesis 29:2025–2034. 10.1093/carcin/bgn168 18632758 10.1093/carcin/bgn168 PMC3202306

[91] Steinauer N, Guo C, Zhang J (2020) The transcriptional corepressor CBFA2T3 inhibits all-trans-retinoic acid-induced myeloid gene expression and differentiation in acute myeloid leukemia. J Biol Chem 295:8887–8900. 10.1074/jbc.RA120.013042 32434928 10.1074/jbc.RA120.013042 PMC7335779

[92] Wu G, Peng J-B (2013) Disease-causing mutations in KLHL3 impair its effect on WNK4 degradation. FEBS Lett 587:1717–1722. 10.1016/j.febslet.2013.04.032 23665031 10.1016/j.febslet.2013.04.032 PMC3697765

[93] Keller KE, Yang Y-F, Sun YY et al (2013) Ankyrin repeat and suppressor of cytokine signaling box containing protein-10 is associated with ubiquitin-mediated degradation pathways in trabecular meshwork cells. Mol Vis 19:1639–1655 23901248 PMC3724959

[94] Narahara S, Sakai E, Kadowaki T et al (2019) KBTBD11, a novel BTB-Kelch protein, is a negative regulator of osteoclastogenesis through controlling Cullin3-mediated ubiquitination of NFATc1. Sci Rep 9:3523. 10.1038/s41598-019-40240-2 30837587 10.1038/s41598-019-40240-2 PMC6401029

[95] Alvarez E, Zhou W, Witta SE, Freed CR (2005) Characterization of the Bex gene family in humans, mice, and rats. Gene 357:18–28. 10.1016/j.gene.2005.05.012 15958283 10.1016/j.gene.2005.05.012

[96] Stratoulias V, Ruiz R, Kanatani S et al (2023) ARG1-expressing microglia show a distinct molecular signature and modulate postnatal development and function of the mouse brain. Nat Neurosci 26:1008–1020. 10.1038/s41593-023-01326-3 37169859 10.1038/s41593-023-01326-3 PMC10244174

[97] Kim HS, Jee SA, Einisadr A et al (2025) Detrimental influence of Arginase-1 in infiltrating macrophages on poststroke functional recovery and inflammatory milieu. Proc Natl Acad Sci U S A 122:e2413484122. 10.1073/pnas.2413484122 39951507 10.1073/pnas.2413484122 PMC11848331

[98] Collmann FM, Pijnenburg R, Schneider G et al (2018) Imaging reporter strategy to monitor gene activation of microglia polarisation states under stimulation. J Neuroimmune Pharmacol 13:371–382. 10.1007/s11481-018-9789-2 29790106 10.1007/s11481-018-9789-2 PMC6096558

[99] von Ehr A, Attaai A, Neidert N et al (2020) Inhibition of microglial TGFβ signaling increases expression of Mrc1. Front Cell Neurosci 14:66. 10.3389/fncel.2020.00066 32296307 10.3389/fncel.2020.00066 PMC7137652

[100] Beltramo E, Mazzeo A, Porta M (2023) Release of pro-inflammatory/angiogenic factors by retinal microvascular cells is mediated by extracellular vesicles derived from M1-activated microglia. Int J Mol Sci 25:15. 10.3390/ijms25010015 38203187 10.3390/ijms25010015 PMC10778795

[101] Canonica J, Foxton R, Garrido MG et al (2023) Delineating effects of angiopoietin-2 inhibition on vascular permeability and inflammation in models of retinal neovascularization and ischemia/reperfusion. Front Cell Neurosci 17:1192464. 10.3389/fncel.2023.1192464 37377777 10.3389/fncel.2023.1192464 PMC10291265

[102] Li L, Jiang W, Yu B et al (2023) Quercetin improves cerebral ischemia/reperfusion injury by promoting microglia/macrophages M2 polarization via regulating PI3K/Akt/NF-κB signaling pathway. Biomed Pharmacother 168:115653. 10.1016/j.biopha.2023.115653 37812891 10.1016/j.biopha.2023.115653

[103] Mirarchi A, Albi E, Arcuri C (2024) Microglia signatures: a cause or consequence of microglia-related brain disorders? Int J Mol Sci 25:10951. 10.3390/ijms252010951 39456734 10.3390/ijms252010951 PMC11507570

[104] Kong G, Liu J, Wang J et al (2025) Engineered extracellular vesicles modified by Angiopep-2 peptide promote targeted repair of spinal cord injury and brain inflammation. ACS Nano 19:4582–4600. 10.1021/acsnano.4c14675 39853366 10.1021/acsnano.4c14675 PMC11803916

[105] Yue F, Cheng Y, Breschi A et al (2014) A comparative encyclopedia of DNA elements in the mouse genome. Nature 515:355–364. 10.1038/nature13992 25409824 10.1038/nature13992 PMC4266106

[106] Panova V, Gogoi M, Rodriguez-Rodriguez N et al (2021) Group-2 innate lymphoid cell-dependent regulation of tissue neutrophil migration by alternatively activated macrophage-secreted Ear11. Mucosal Immunol 14:26–37. 10.1038/s41385-020-0298-2 32457448 10.1038/s41385-020-0298-2 PMC7790759

[107] Ong EZ, Koh CWT, Tng DJH et al (2023) RNase2 is a possible trigger of acute-on-chronic inflammation leading to mRNA vaccine-associated cardiac complication. Med 4:353-360.e2. 10.1016/j.medj.2023.04.001 37105176 10.1016/j.medj.2023.04.001 PMC10131284

[108] Okazaki Y, Furuno M, Kasukawa T et al (2002) Analysis of the mouse transcriptome based on functional annotation of 60,770 full-length cDNAs. Nature 420:563–573. 10.1038/nature01266 12466851 10.1038/nature01266

[109] An N, Blumer JB, Bernard ML, Lanier SM (2008) The PDZ and band 4.1 containing protein Frmpd1 regulates the subcellular location of activator of G-protein signaling 3 and its interaction with G-proteins. J Biol Chem 283:24718–24728. 10.1074/jbc.M803497200 18566450 10.1074/jbc.M803497200 PMC2529004

[110] Campla CK, Bocchero U, Strickland R et al (2022) Frmpd1 facilitates trafficking of G-protein transducin and modulates synaptic function in rod photoreceptors of mammalian retina. eNeuro 9:ENEURO.0348-22.2022. 10.1523/ENEURO.0348-22.2022 36180221 10.1523/ENEURO.0348-22.2022 PMC9581579

[111] Chung W-S, Clarke LE, Wang GX et al (2013) Astrocytes mediate synapse elimination through MEGF10 and MERTK pathways. Nature 504:394–400. 10.1038/nature12776 24270812 10.1038/nature12776 PMC3969024

[112] Ahmed S, Polis B, Jamwal S et al (2024) Transient impairment in microglial function causes sex-specific deficits in synaptic maturity and hippocampal function in mice exposed to early adversity. Brain Behav Immun 122:95–109. 10.1016/j.bbi.2024.08.010 39134183 10.1016/j.bbi.2024.08.010 PMC11402597

[113] Kioka N, Ueda K, Amachi T (2002) Vinexin, CAP/ponsin, ArgBP2: a novel adaptor protein family regulating cytoskeletal organization and signal transduction. Cell Struct Funct 27:1–7. 10.1247/csf.27.1 11937713 10.1247/csf.27.1

[114] Wen K-X, Miliç J, El-Khodor B et al (2016) The role of DNA methylation and histone modifications in neurodegenerative diseases: a systematic review. PLoS ONE 11:e0167201. 10.1371/journal.pone.0167201 27973581 10.1371/journal.pone.0167201 PMC5156363

[115] Bersani I, Ronci S, Savarese I et al (2024) COL4A1 gene mutations and perinatal intracranial hemorrhage in neonates: case reports and literature review. Front Pediatr 12:1417873. 10.3389/fped.2024.1417873 38978838 10.3389/fped.2024.1417873 PMC11228817

[116] Remme CA, Bezzina CR (2010) Sodium channel (dys)function and cardiac arrhythmias. Cardiovasc Ther 28:287–294. 10.1111/j.1755-5922.2010.00210.x 20645984 10.1111/j.1755-5922.2010.00210.x

[117] Remme CA (2023) SCN5A channelopathy: arrhythmia, cardiomyopathy, epilepsy and beyond. Philos Trans R Soc Lond B Biol Sci 378:20220164. 10.1098/rstb.2022.0164 37122208 10.1098/rstb.2022.0164 PMC10150216

[118] Labib D, Wang Z, Prakash P et al (2022) Proteomic alterations and novel markers of neurotoxic reactive astrocytes in human induced pluripotent stem cell models. Front Mol Neurosci 15:870085. 10.3389/fnmol.2022.870085 35592112 10.3389/fnmol.2022.870085 PMC9113221

[119] Wang J, Wang L, Wu Q et al (2024) Interleukin-4 modulates neuroinflammation by inducing phenotypic transformation of microglia following subarachnoid hemorrhage. Inflammation 47:390–403. 10.1007/s10753-023-01917-z 37898992 10.1007/s10753-023-01917-z PMC10799105

[120] Ransohoff RM (2016) How neuroinflammation contributes to neurodegeneration. Science 353:777–783. 10.1126/science.aag2590 27540165 10.1126/science.aag2590

[121] Masuda T, Sankowski R, Staszewski O, Prinz M (2020) Microglia heterogeneity in the single-cell era. Cell Rep 30:1271–1281. 10.1016/j.celrep.2020.01.010 32023447 10.1016/j.celrep.2020.01.010

[122] Thakur S, Dhapola R, Sarma P et al (2023) Neuroinflammation in Alzheimer’s disease: current progress in molecular signaling and therapeutics. Inflammation 46:1–17. 10.1007/s10753-022-01721-1 35986874 10.1007/s10753-022-01721-1

[123] Akhmetzyanova E, Kletenkov K, Mukhamedshina Y, Rizvanov A (2019) Different approaches to modulation of microglia phenotypes after spinal cord injury. Front Syst Neurosci 13:37. 10.3389/fnsys.2019.00037 31507384 10.3389/fnsys.2019.00037 PMC6718713

[124] Maguire E, Connor-Robson N, Shaw B et al (2022) Assaying microglia functions in vitro. Cells 11:3414. 10.3390/cells11213414 36359810 10.3390/cells11213414 PMC9654693

[125] Jiang J, Tang B, Wang L et al (2022) Systemic LPS-induced microglial activation results in increased GABAergic tone: a mechanism of protection against neuroinflammation in the medial prefrontal cortex in mice. Brain Behav Immun 99:53–69. 10.1016/j.bbi.2021.09.017 34582995 10.1016/j.bbi.2021.09.017

[126] Guedes JR, Ferreira PA, Costa J et al (2023) IL-4 shapes microglia-dependent pruning of the cerebellum during postnatal development. Neuron 111:3435-3449.e8. 10.1016/j.neuron.2023.09.031 37918358 10.1016/j.neuron.2023.09.031

[127] Jung H, Lee D, You H et al (2023) LPS induces microglial activation and GABAergic synaptic deficits in the hippocampus accompanied by prolonged cognitive impairment. Sci Rep 13:6547. 10.1038/s41598-023-32798-9 37085584 10.1038/s41598-023-32798-9 PMC10121592

[128] Mishra MK, Rawji KS, Keough MB et al (2021) Harnessing the benefits of neuroinflammation: generation of macrophages/microglia with prominent remyelinating properties. J Neurosci 41:3366–3385. 10.1523/JNEUROSCI.1948-20.2021 33712513 10.1523/JNEUROSCI.1948-20.2021 PMC8051677

[129] Sanford SAI, McEwan WA (2022) Type-I interferons in Alzheimer’s disease and other tauopathies. Front Cell Neurosci 16:949340. 10.3389/fncel.2022.949340 35910253 10.3389/fncel.2022.949340 PMC9334774

[130] Nazmi A, Field RH, Griffin EW et al (2019) Chronic neurodegeneration induces type i interferon synthesis via STING, shaping microglial phenotype and accelerating disease progression. Glia 67:1254–1276. 10.1002/glia.23592 30680794 10.1002/glia.23592 PMC6520218

[131] Muzio L, Viotti A, Martino G (2021) Microglia in neuroinflammation and neurodegeneration: from understanding to therapy. Front Neurosci 15:742065. 10.3389/fnins.2021.742065 34630027 10.3389/fnins.2021.742065 PMC8497816

[132] Holtman IR, Raj DD, Miller JA et al (2015) Induction of a common microglia gene expression signature by aging and neurodegenerative conditions: a co-expression meta-analysis. Acta Neuropathol Commun 3:31. 10.1186/s40478-015-0203-5 26001565 10.1186/s40478-015-0203-5 PMC4489356

[133] Grabert K, Michoel T, Karavolos MH et al (2016) Microglial brain region-dependent diversity and selective regional sensitivities to aging. Nat Neurosci 19:504–516. 10.1038/nn.4222 26780511 10.1038/nn.4222 PMC4768346

[134] Bohlen CJ, Bennett FC, Tucker AF et al (2017) Diverse requirements for microglial survival, specification, and function revealed by defined-medium cultures. Neuron 94:759-773.e8. 10.1016/j.neuron.2017.04.043 28521131 10.1016/j.neuron.2017.04.043 PMC5523817

