# Correction: Sustained Transcriptional Response to Lipopolysaccharide and Interleukin-4 in an Immortalized Mouse Microglial Cell Line

**DOI:** 10.1007/s12035-026-05765-4

**Published:** 2026-02-26

**Authors:** Amanda Herrero-González, Alba Puente-Sanz, Diego Pérez-Rodríguez, Berta Anuncibay-Soto, Michal Letek, Marta Regueiro-Purriños, Arsenio Fernández-López

**Affiliations:** 1 Departamento de Biología Molecular, Universidad de León, León, Spain; 2 Neural Therapies S.L., Edificio Institutos de Investigación, Local B43. Campus de Vegazana, León, Spain; 3 Dipartimento Di Scienze Della Salute, Universitá Di Firenze, Florence, Italy; 4 Department of Clinical and Movement Neurosciences, UCL Queen Square Institute of Neurology, London, UK; 5 Department of Life Sciences, Imperial College London (ICL), London, UK; 6 Departamento de Medicina, Cirugía y Anatomía Veterinaria, Universidad de León, León, Spain

**Correction: Molecular Neurobiology (2026) 63:425**

10.1007/s12035-026-05711-4

In the original version of this article, the given and family names of authors were incorrectly structured. These names should be written as "Amanda Herrero-González, Alba Puente-Sanz, Diego Pérez-Rodríguez, Berta Anuncibay-Soto, Michal Letek, Marta Regueiro-Purriños, Arsenio Fernández-López".

The original article has been corrected.

