# Supplementary material for: Sustained Transcriptional Response to Lipopolysaccharide and Interleukin-4 in an Immortalized Mouse Microglial Cell Line
Source: Mol Neurobiol. 2026 Feb 6;63(1):425. doi: 10.1007/s12035-026-05711-4 (PMC12881133; doi:10.1007/s12035-026-05711-4)
Supplement: Supplementary file 1 — (DOCX 112 KB) [file 12035_2026_5711_MOESM1_ESM.docx]

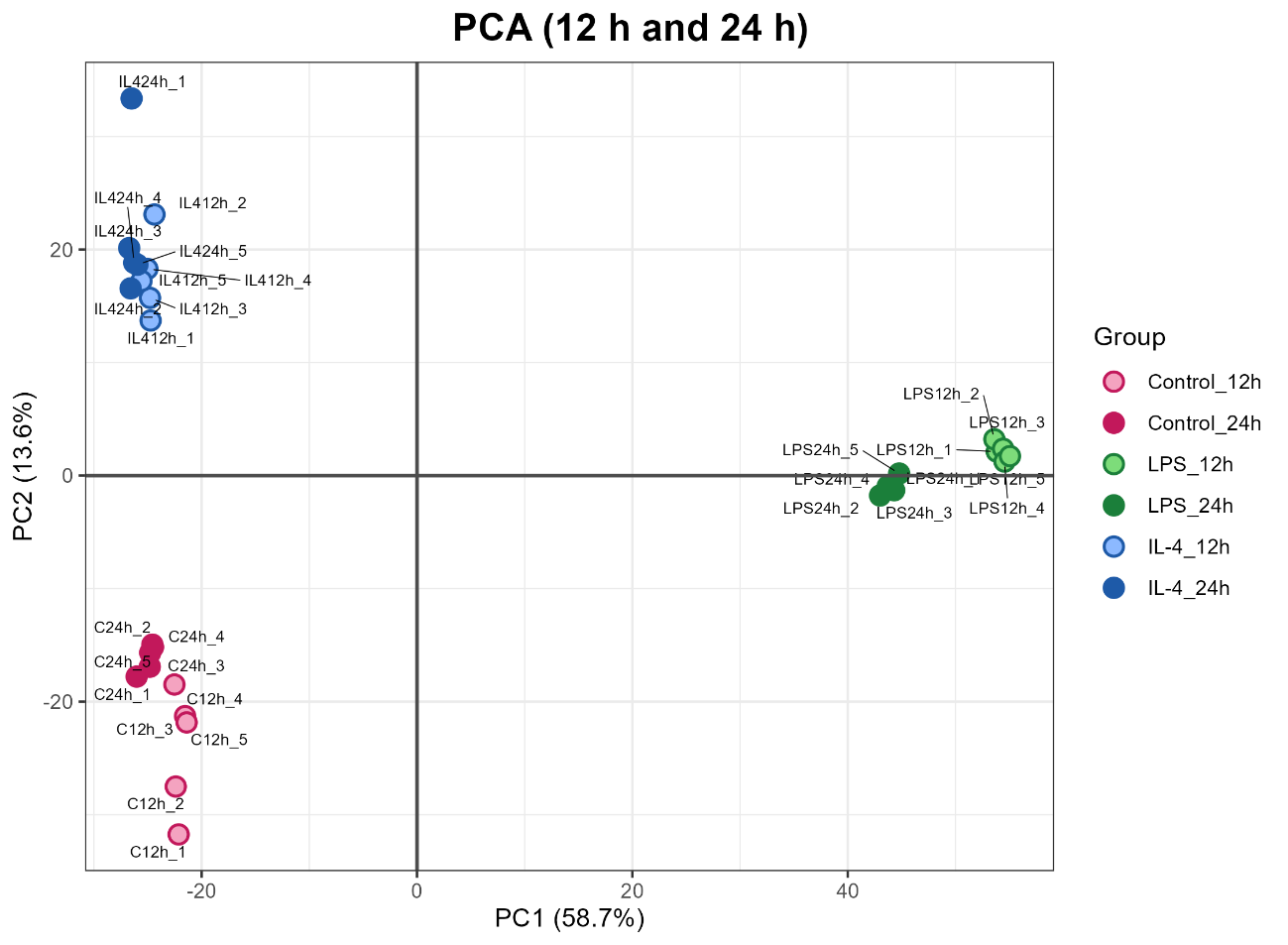


**Supplementary Figure 1. Principal Component Analysis (PCA) of RNA-seq samples**. PCA was performed on variance-stabilized (VST) gene-level counts (DESeq2) from control, LPS- and IL-4–treated samples collected at 12 h and 24 h. The first two principal components are shown; PC1 and PC2 explain 58.7% and 13.6% of the total variance, respectively (cumulative variance, 72.3%). Each point represents one biological replicate, labeled with its sample identifier.
